# Comparative mitogenome analyses uncover mitogenome features and phylogenetic implications of the subfamily Cobitinae

**DOI:** 10.1186/s12864-020-07360-w

**Published:** 2021-01-14

**Authors:** Peng Yu, Li Zhou, Wen-Tao Yang, Li-jun Miao, Zhi Li, Xiao-Juan Zhang, Yang Wang, Jian-Fang Gui

**Affiliations:** 1 State Key Laboratory of Freshwater Ecology and Biotechnology, Institute of Hydrobiology, the Innovation Academy of Seed Design, Chinese Academy of Sciences, Wuhan, 430072 China; 2 University of Chinese Academy of Sciences, Beijing, 100049 China

**Keywords:** Cobitinae, Loach, Mitochondrial genome, mtDNA introgression, Phylogeny, Divergence time

## Abstract

**Background:**

Loaches of Cobitinae, widely distributed in Eurasian continent, have high economic, ornamental and scientific value. However, the phylogeny of Cobitinae fishes within genera or family level remains complex and controversial. Up to now, about 60 Cobitinae mitogenomes had been deposited in GenBank, but their integrated characteristics were not elaborated.

**Results:**

In this study, we sequenced and analyzed the complete mitogenomes of a female *Cobits macrostigma*. Then we conducted a comparative mitogenome analysis and revealed the conserved and unique characteristics of 58 Cobitinae mitogenomes, including *C. macrostigma*. Cobitinae mitogenomes display highly conserved tRNA secondary structure, overlaps and non-coding intergenic spacers. In addition, distinct base compositions were observed among different genus and significantly negative linear correlation between AT% and AT-skew were found among Cobitinae, genus *Cobitis* and *Pangio* mitogenomes, respectively. A specific 3 bp insertion (GCA) in the *atp8*-*atp6* overlap was identified as a unique feature of loaches, compared to other Cypriniformes fish. Additionally, all protein coding genes underwent a strong purifying selection. Phylogenetic analysis strongly supported the paraphyly of *Cobitis* and polyphyly of *Misgurnus*. The strict molecular clock predicted that Cobitinae might have split into northern and southern lineages in the late Eocene (42.11 Ma), furthermore, mtDNA introgression might occur (14.40 Ma) between ancestral species of *Cobitis* and ancestral species of *Misgurnus*.

**Conclusions:**

The current study represents the first comparative mitogenomic and phylogenetic analyses within Cobitinae and provides new insights into the mitogenome features and evolution of fishes belonging to the cobitinae family.

**Supplementary Information:**

The online version contains supplementary material available at 10.1186/s12864-020-07360-w.

## Background

Vertebrate mitogenome is a small (16–17 kb) and circular double-stranded molecule [1]. It contains 37 genes including 22 tRNA genes, 13 PCGs and two rRNA genes [1]. It also has two noncoding regions, O_L_ and CR, and the latter contains regulatory elements for controlling the transcription and replication of mtDNA molecule [2, 3]. Due to its unique features, such as high copy numbers in tissues, simple genomic organization, maternal inheritance, almost unambiguous orthology, haploid inheritance and high nucleotide substitution rate [4–6], mitogenome has been widely applied in species identification, i.e., DNA barcoding, as well as population genetics, conservation biology, molecular phylogenetics and evolutionary processes [7–13]. Gene arrangements of fish mitogenomes are generally conserved, only with a few exceptions [1]. However, the genome sequence length, the bias of base composition and start/stop codon, the overlap and IGSs are diverse among different species [14].

Cobitinae is a subfamily of Cobitidae that was first identified by Hora (1932). To date, it contains 214 species recorded in FishBase, covering 21 genera, such as *Cobits*, *Misgurnus* and *Paramisgurnus* [15]. Loaches of subfamily Cobitinae are bottom-dwelling fishes and widely distributed in Eurasian continent. They usually possess high economic, ornamental and scientific research value. Loach commercial farming, including cobitid loach (*M. anguillicaudatus*) and large-scale loach (*P. dabryanus*), occupies a significant position in freshwater aquaculture of Asia, due to their enjoyable taste, high nutritional value, rapid growth and strong adaptation [16–18]. In China, loach is used as a diet therapy or folk remedy for patient’s recovery or treatment of many diseases, such as hepatitis, osteomyeitis, carbuncles, and cancers. Many *Cobitis* populations are mixed diploid-polyploid, even bisexual and unisexual forms co-existing in the same niche [19–21]. They are suitable as models to reveal the relationship among hybridization, polyploidization, reproduction, speciation and evolution [21–23]. Due to their great diversity, they are also used to trace the biogeographic history of freshwater systems and to reflect geologic events [24]. Cobitinae fishes usually inhabit various benthic habitats in rivers, lakes, streams and ponds [25]. However, dilapidation of the ecological environment has led to a decrease of benthic organisms [26, 27]. Cobitinae fishes are seriously threatened and their wild populations are gradually decreasing [28]. On this account, the diversity of these benthic fishes have been used as a bioindicator to assess the quality of the ecological environment [29, 30]. In addition, many Cobitinae species, such as the “kuhli loaches”, are well-known in Southeast Asia and Europe as ornamental fish for their varied morphological patterns and the ability to ingest bottom organic residues.

Cobitinae fishes are difficult to be classified because of their morphological similarity and high plasticity in morphology [31]. Although the secondary sexual dimorphism is used to define genera, it is not always congruent with the current genera definitions. The molecular phylogeny of Cobitinae fishes has been studied at the genera or family level via one or two mitochondrial and/or nuclear genes [24, 31–36], and remains complex and controversial. For example, based on mitochondrial gene *cytb* and nuclear gene *rag-1*, Perdices et al. (2016) [37] reconstructed the phylogenetic relationship of Northern Clade of family Cobitidae that inhabit in Europe, and North and Northwest parts of Asia. The subfamily Cobitinae was divided into *Cobitis* sensu lato group (*Cobitis*, *Iksookimia*, *Niwaella* and *Kichulchoia*), *Misgurnus* sensu lato group (*Misgurnus*, *Paramisgurnus* and *Koreocobitis*), *Microcobitis*, and *Sabanejewia*. Although the monophyly of the groups were resolved, the relationships within the groups are discordant with current taxonomic status.

Up to now, about 60 mitogenomes, covering more than 40 species of Cobitinae, have been deposited into GenBank [38–55]. Although a few mitogenomes characteristics were described, the integrated characteristics of Cobitinae mitogenomes are still not well known. In this study, we sequenced the mitogenome of *C. macrostigma*, the type species of the genus *Cobitis* [25], and compared it with other 41 species (57 individuals) to amplify detailed features of the Cobitinae mitogenomes. Additionally, we assembled a large sequence matrix (11,442 bp) of 58 Cobitinae mitogenomes and two outgroups to investigate the phylogenetic status and the origin time of Cobitinae fishes.

## Results

### General features of *C. macrostigma* mitogenome

**Fig. 1:**
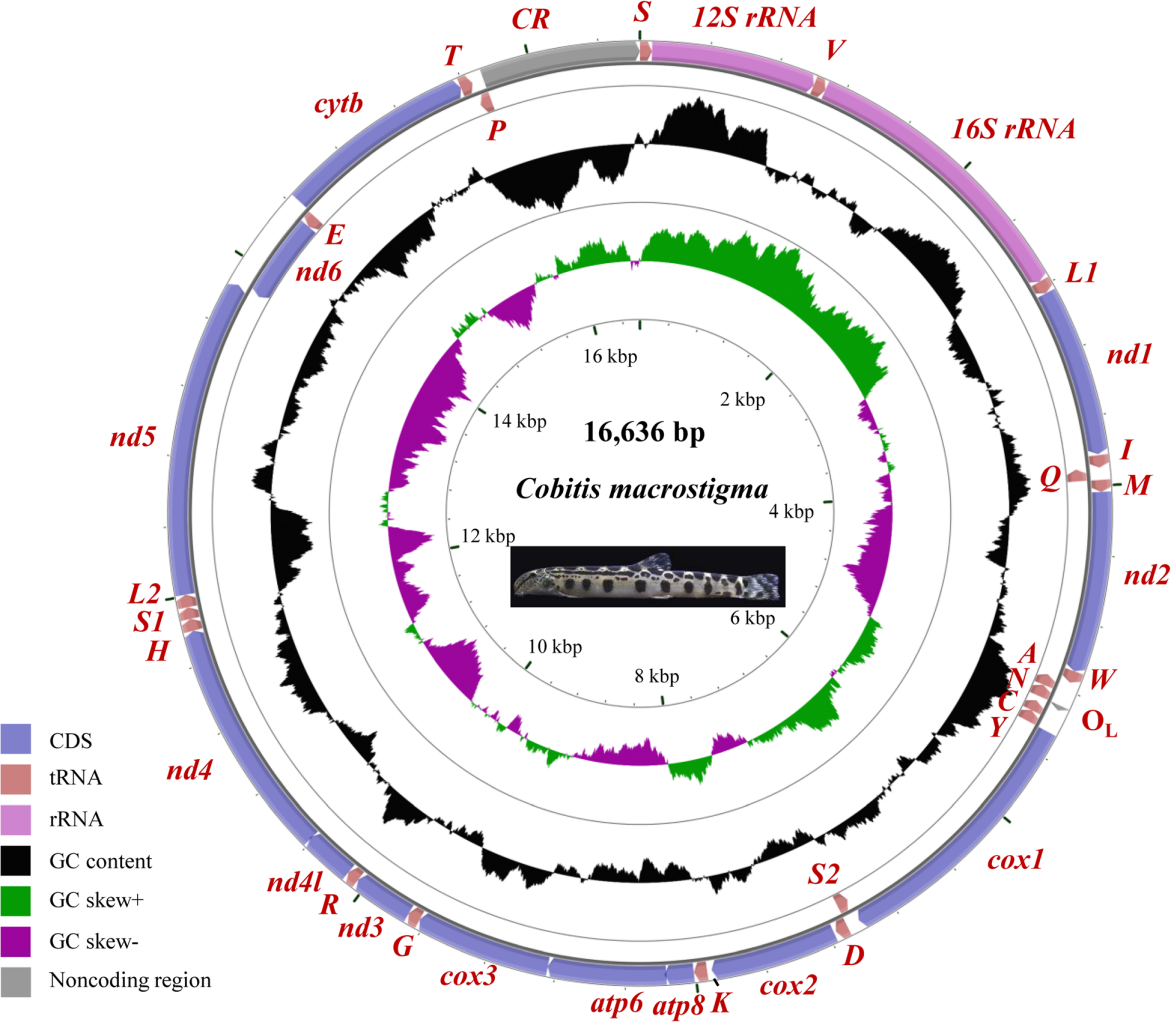
Circular sketch map of the *C. macrostigma* mitogenome. Different colors represent different gene blocks

The mitogenome of *C. macrostigma* was sequenced, annotated and compared with 57 Cobitinae mitogenomes (Table 1). It contains 13 PCGs (*nd1–6*, *nd4l*, *cox1–3*, *cytb*, *atp6* and *atp8*), 22 tRNA genes, two rRNA genes (*12S rRNA* and *16S rRNA*) and two non-coding regions (O_L_ and CR) (GenBank: MT259034). Gene order and orientation are same to most teleost mitogenomes (Fig. 1, Table 2). PCGs range from 168 bp (*atp8*) to 1551 bp (*cox1*) in size, with a total length of 11,427 bp. tRNAs vary from 66 bp (*tRNA*^*Cys*^(*C*)) to 76 bp (*tRNA*^*Lys*^(*K*)) in size, with a total length of 1557 bp. The length of small encoding subunit *12S rRNA* and large subunit *16S rRNA* are 952 bp and 1675 bp, respectively. They are flanked by *tRNA*^*Phe*^ and *tRNA*^*Leu(UUR)*^ and interposed by *tRNA*^*Val*^. Among 58 mitogenomes analyzed, the entire mitogenome of *C. macrostigma* has the highest (99.6%) similarity with *C. granoei* and lowest (88.2%) with *C. sinensis*.

**Table 1:** Species, GenBank accession number and length of mitogenomes used in this study

	Genus	Species	Accession ID	Sequence length (bp)	Reference
1	*Cobitis*	*Cobitis macrostigma*	MT259034	16,636	this study
2	*Acantopsis*	*Acantopsis choirorhynchos*	AB242161.1	16,600	[38]
3	*Acanthopsoides*	*Acanthopsoides gracilentus*	NC_029438.1	16,603	Unpublished
4	*Canthophrys*	*Canthophrys gongota*	NC_031576.1	16,561	Unpublished
5	*Cobitis*	*Cobitis biwae*	NC_027663.1	16,642	[39]
6	*Cobitis*	*Cobitis choii*	NC_010649.2	16,566	[40]
7	*Cobitis*	*Cobitis elongatoides*	NC_023947.1	16,541	[41]
8	*Cobitis*	*Cobitis granoei*	NC_023473.1	16,636	[42]
9	*Cobitis*	*Cobitis lutheri*	NC_022717.1	16,639	Unpublished
10	*Cobitis*	*Cobitis minamorii minamorii*	AP013309.1	16,645	Unpublished
11	*Cobitis*	*Cobitis matsubarai*	NC_029441.1	16,636	Unpublished
12	*Cobitis*	*Cobitis nalbanti*	MH349461.1	16,631	[43]
13	*Cobitis*	*Cobitis sp. (1)*	AP013307.1	16,571	Unpublished
14	*Cobitis*	*Cobitis sp. (2)*	AP013306.1	16,570	Unpublished
15	*Cobitis*	*Cobitis sp. (3)*	AP013296.1	16,576	Unpublished
16	*Cobitis*	*Cobitis striata (1)*	AP010782.1	16,646	[44]
17	*Cobitis*	*Cobitis striata (2)*	AB054125.1	16,572	[45]
18	*Cobitis*	*Cobitis striata striata*	AP013311.1	16,631	Unpublished
19	*Cobitis*	*Cobitis sinensis*	NC_007229.1	16,553	Unpublished
20	*Cobitis*	*Cobitis takatsuensis (1)*	AP009306.1	16,647	[44]
21	*Cobitis*	*Cobitis takatsuensis (2)*	AP011290.1	16,578	[39]
22	*Iksookimia*	*Iksookimia longicorpa*	NC_027850.1	16,624	Unpublished
23	*Kichulchoia*	*Kichulchoia multifasciata*	AP011337.1	16,643	Unpublished
24	*Koreocobitis*	*Koreocobitis naktongensis*	HM535625.1	16,567	Unpublished
25	*Kottelatlimia*	*Kottelatlimia pristes*	NC_031597.1	16,588	Unpublished
26	*Lepidocephalichthys*	*Lepidocephalichthys annandalei*	AP013313.1	16,337	Unpublished
27	*Lepidocephalichthys*	*Lepidocephalichthys guntea*	NC_031593.1	16,567	Unpublished
28	*Lepidocephalichthys*	*Lepidocephalichthys hasselti*	AP013334.1	15,897	Unpublished
29	*Lepidocephalichthys*	*Lepidocephalichthys micropogon*	NC_031595.1	16,608	Unpublished
30	*Lepidocephalichthys*	*Lepidocephalichthys sp.*	AP013314.1	15,917	Unpublished
31	*Lepidocephalus*	*Lepidocephalus macrochir*	NC_031596.1	16,556	Unpublished
32	*Misgurnus*	*Misgurnus anguillicaudatus (1)*	KC823274.1	16,646	[46]
33	*Misgurnus*	*Misgurnus anguillicaudatus (2)*	KM186181.1	16,645	Unpublished
34	*Misgurnus*	*Misgurnus anguillicaudatus (3)*	KC881110.1	16,643	[47]
35	*Misgurnus*	*Misgurnus anguillicaudatus (4)*	KC734881.1	16,643	[48]
36	*Misgurnus*	*Misgurnus anguillicaudatus (5)*	KC884745.1	16,644	[47]
37	*Misgurnus*	*Misgurnus anguillicaudatus (6)*	MG938590.1	16,646	Unpublished
38	*Misgurnus*	*Misgurnus anguillicaudatus (7)*	KC509900.1	16,646	[49]
39	*Misgurnus*	*Misgurnus anguillicaudatus (8)*	MF579257.1	16,647	Unpublished
40	*Misgurnus*	*Misgurnus anguillicaudatus (9)*	KC509901.1	16,646	[49]
41	*Misgurnus*	*Misgurnus anguillicaudatus (10)*	KC762740.1	16,645	[46]
42	*Misgurnus*	*Misgurnus anguillicaudatus (11)*	HM856629.1	16,634	[50]
43	*Misgurnus*	*Misgurnus anguillicaudatus (12)*	AP011291.1	16,641	[39]
44	*Misgurnus*	*Misgurnus anguillicaudatus (13)*	DQ026434.1	16,565	[51]
45	*Misgurnus*	*Misgurnus anguillicaudatus (14)*	NC_011209.1	16,565	[51]
46	*Misgurnus*	*Misgurnus bipartitus*	NC_022854.1	16,636	[52]
47	*Misgurnus*	*Misgurnus mizolepis*	NC_038151.1	16,571	Unpublished
48	*Misgurnus*	*Misgurnus mohoity*	KF386025.1	16,566	[53]
49	*Misgurnus*	*Misgurnus nikolskyi*	AB242171.1	16,570	[38]
50	*Niwaella*	*Niwaella delicata*	AP009308.1	16,571	[44]
51	*Paramisgurnus*	*Paramisgurnus dabryanus (1)*	KR349175.1	16,570	[54]
52	*Paramisgurnus*	*Paramisgurnus dabryanus (2)*	AP012124.1	16,571	[39]
53	*Paramisgurnus*	*Paramisgurnus dabryanus (3)*	KJ027397.1	16,570	Unpublished
54	*Pangio*	*Pangio anguillaris*	AB242168.1	16,602	[38]
55	*Pangio*	*Pangio cuneovirgata*	NC_031594.1	16,596	Unpublished
56	*Pangio*	*Pangio kuhlii*	NC_031599.1	16,601	Unpublished
57	*Pangio*	*Pangio oblonga*	NC_031592.1	16,600	Unpublished
58	*Microcobitis*	*Microcobitis sp.*	AP013297.1	16,549	Unpublished
59	*Sinorhodeus*	*Sinorhodeus microlepis*	MH190825	16,591	[15]
60	*Rhodeus*	*Rhodeus shitaiensis*	KF176560.1	16,774	[55]

**Table 2:** Annotation of the *C. macrostigma* mitogenome

Feature	Position	Nucleotide size (bp)	Start codon	Stop codon	Amino acid	Anti-codon	Intergenic nucleotide^a^	Strand^b^
*tRNA*^*Phe*^ *(S)*	1–69	69				GAA	0	H
*12S rRNA*	70–1021	952					0	H
*tRNA*^*Val*^ *(V)*	1022–1093	72				TAC	0	H
*16S rRNA*	1094–2768	1675					0	H
*tRNA*^*Leu(UUR)*^ *(L1)*	2769–2843	75				TAA	1	H
*nd1*	2845–3819	975	ATG	TAA	324		6	H
*tRNA*^*Ile*^ *(I)*	3826–3897	72				GAT	-2	H
*tRNA*^*Gln*^ *(Q)*	3896–3966	71				TTG	1	L
*tRNA*^*Met*^ *(M)*	3968–4036	69				CAT	0	H
*nd2*	4037–5081	1045	ATG	T	348		0	H
*tRNA*^*Trp*^ *(W)*	5082–5151	70				TCA	1	H
*tRNA*^*Ala*^ *(A)*	5153–5221	69				TGC	1	L
*tRNA*^*Asn*^ *(N)*	5223–5295	73				GTT	0	L
L-strand replication origin (O_L_)	5296–5325	30					0	
*tRNA*^*Cys*^ *(C)*	5326–5391	66				GCA	0	L
*tRNA*^*Tyr*^ *(Y)*	5392–5460	69				GTA	1	L
*cox1*	5462–7012	1551	GTG	TAA	516		1	H
*tRNA*^*Ser(UCN)*^ *(S2)*	7014–7084	71				TGA	2	L
*tRNA*^*Asp*^ *(D)*	7087–7158	72				GTC	13	H
*cox2*	7172–7906	735	ATG	TAA	244		26	H
*tRNA*^*Lys*^ *(K)*	7933–8008	76				TTT	1	H
*atp8*	8010–8177	168	ATG	TAA	55		−10	H
*atp6*	8168–8851	684	ATG	TAA	227		−1	H
*cox3*	8851–9634	784	ATG	T	261		0	H
*tRNA*^*Gly*^ *(G)*	9635–9706	72				TCC	0	H
*nd3*	9707–10,055	349	ATG	T	116		0	H
*tRNA*^*Arg*^ *(R)*	10,056–10,125	70				TCG	0	H
*nd4l*	10,126–10,422	297	ATG	TAA	98		−7	H
*nd4*	10,416–11,797	1382	ATG	TA	460		0	H
*tRNA*^*His*^ *(H)*	11,798–11,866	69				GTG	0	H
*tRNA*^*Ser(AGY)*^ *(S1)*	11,867–11,934	68				GCT	1	H
*tRNA*^*Leu(CUN)*^ *(L2)*	11,936–12,008	73				TAG	0	H
*nd5*	12,009–13,847	1839	ATG	TAG	612		−4	H
*nd6*	13,844–14,365	522	ATG	TAA	173		0	L
*tRNA*^*Glu*^ *(E)*	14,366–14,434	69				TTC	6	L
*cytb*	14,441–15,581	1141	ATG	T	380		0	H
*tRNA*^*Thr*^ *(T)*	15,582–15,653	72				TGT	−2	H
*tRNA*^*Pro*^ *(P)*	15,652–15,721	70				TGG	−2	L
Control region (CR)	15,720–16,636	917						

### Highly conserved tRNAs secondary structure, overlaps and non-coding intergenic spacers among Cobitinae mitogenomes

**Fig. 2:**
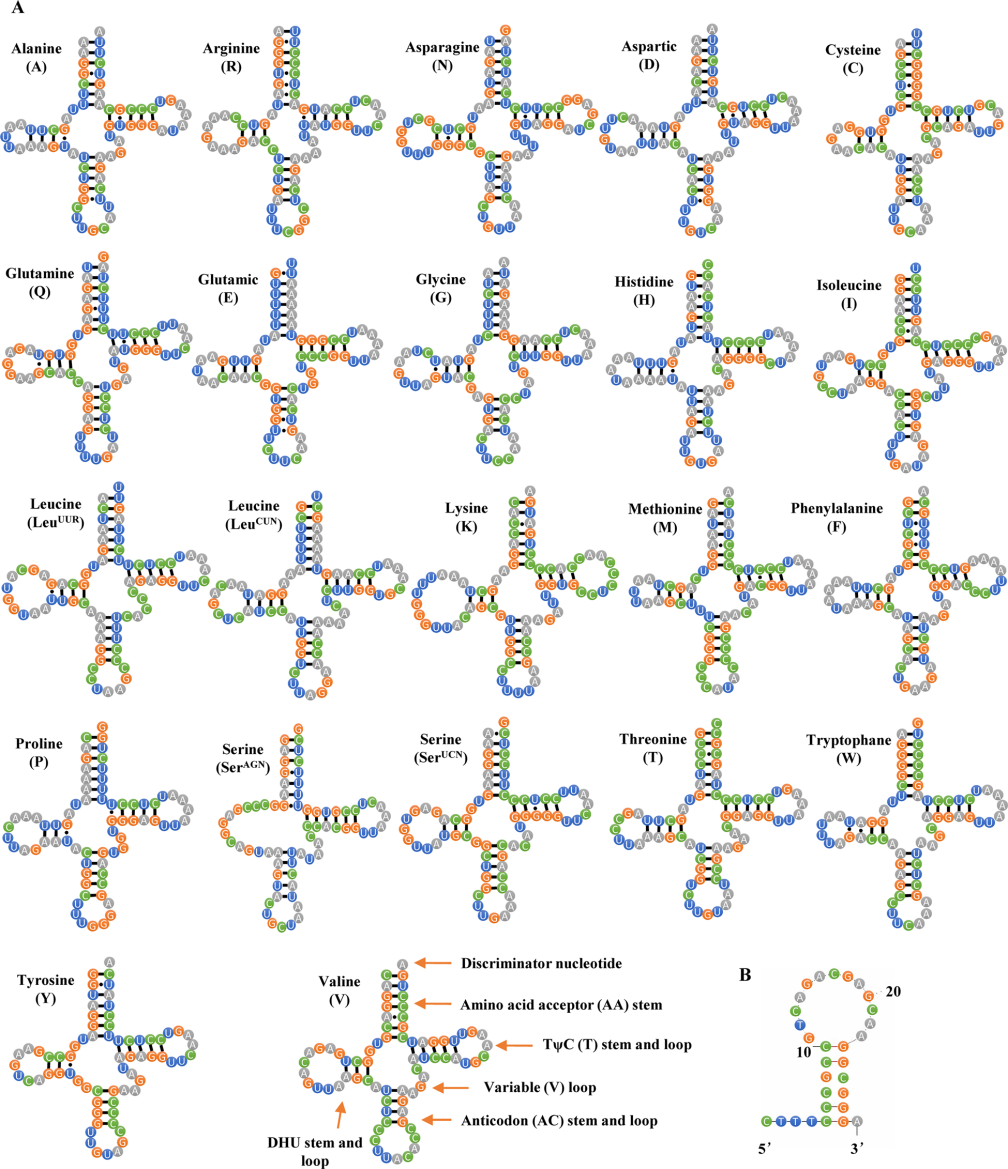
Putative secondary structure of tRNAs (**a**) and O_L_ (**b**) of Cobitinae mitogeneomes. *C. macrostigma* mitogenome is taken as an example. tRNAs are labeled with their corresponding amino acids. Dashes (−) indicate Watson–Crick bonds, and dots (·) indicate mispaired nucleotide bonds

Cobitinae mitogenomes range from 16,337 bp (*L. annandalei*) to 16,647 bp (*M. anguillicaudatus* and *C. takatsuensis*) in length (Table 1). Their gene composition, gene arrangement and strand bias are highly conserved (Fig.1 and Table 2). Among the 22 tRNAs, due to the absence of DHU arm, *tRNA*^*ser(AGN)*^ (*S1*) is the only one that is not folded into the typical clover-leaf secondary structure (Fig. 2a). In the Cobitinae mitogenomes, unmatched base pairs are widespread among tRNAs. Taking *C. macrostigma* as an example, there are 446 base pairs among the 22 tRNAs, and only one gene (*tRNA*^*Leu(CUN)*^) possesses a fully paired stem. In the 425 base pairs of other 21 tRNAs, there are 43 (10.1%) unmatched base pairs that contain 28 noncanonical matches of G-U and 15 other mismatches, including A-C (7), A-A (1), C-C (2), C-U (2), and U-U (3) (Fig. 2a). Most of them are located in the acceptor, DHU and anticodon stems.

**Fig. 3:**
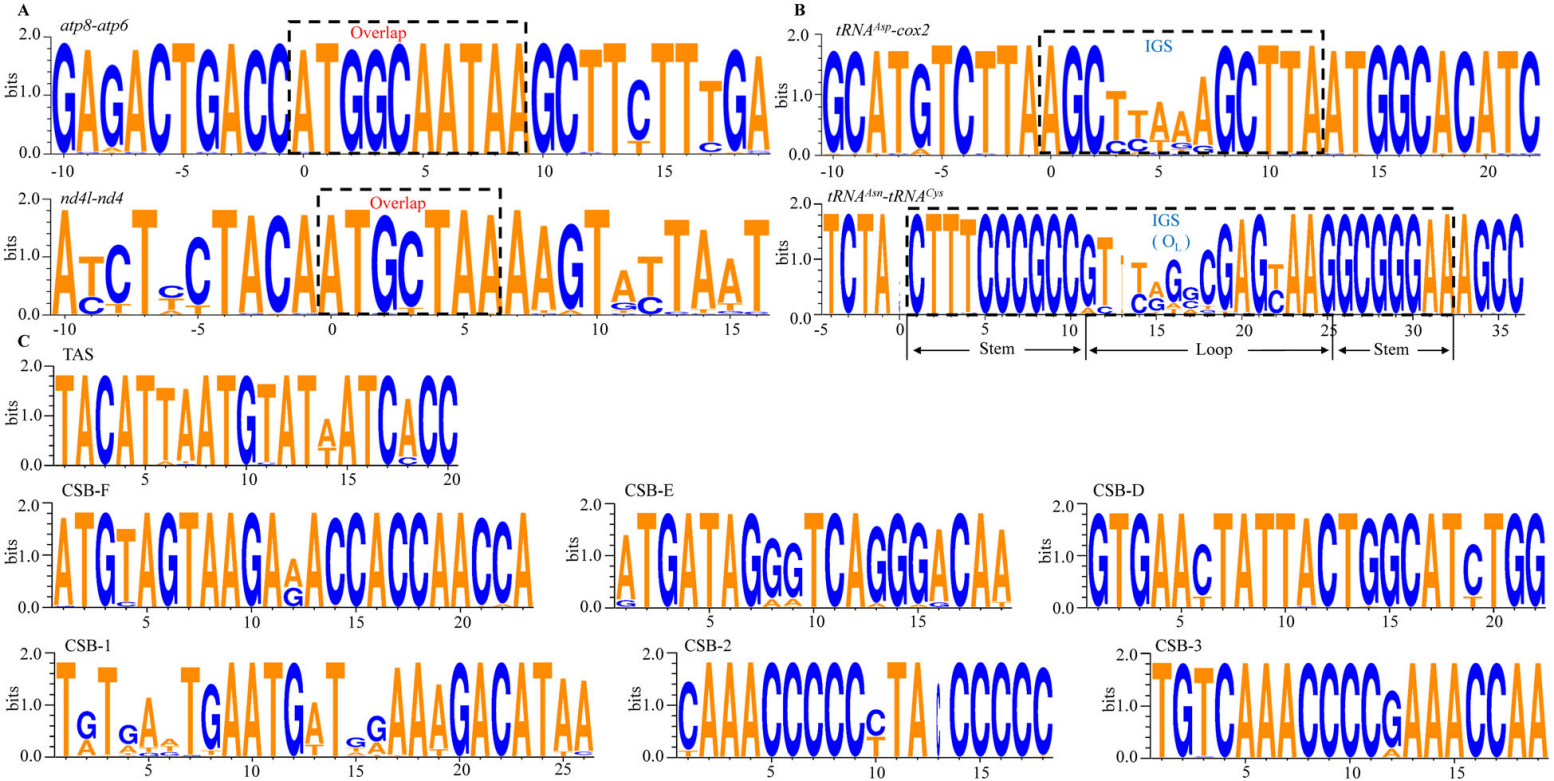
Sequence logo of gene overlaps in *atp8*-*atp6* (**a**), non-coding intergenic spacers in *tRNA*^*Asp*^-*cox2* (**b**) and short conserved motif in CR of Cobitinae mitogenomes (**c**)

We also compared the gene overlaps and IGSs among 58 Cobitinae mitogenomes. Two long overlaps (*atp8*-*atp6* and *nd4l*-*nd4*) and two long IGSs (O_L_ and *tRNA*^*Asp*^-*cox2*) were found in Cobitinae mitogenomes. Highly conserved motifs “ATGCTAA” and “ATGGCAATAA” were found in the overlapped junctions between *nd4l* and *nd4*, and between *atp8* and *atp6,* respectively (Fig. 3a). There are also several small overlaps between adjacent tRNA genes, such as *tRNA*^*Ile*^ - *tRNA*^*Gln*^ and *tRNA*^*Thr*^ - *tRNA*^*Pro*^. O_L_ is located within the five gene cluster (WANCY) (Table 2, Fig.1) and its secondary structure shows a stable stem-loop hairpin, which is strengthened by six C-G base pairs (Fig. 2b). Among the 31 bp of O_L_, the C-G base pairs on stems are highly conserved while the loops in the middle are variable (Fig. 3b). Another long IGS, between *tRNA*^*Asp*^ and *cox2*, is also conserved in the 5′ and 3′ end, and highly variable in the middle.

CR, located between *tRNA*^*Pro*^ and *tRNA*^*Phe*^, is the most variable region in Cobitinae mitogenomes and ranges from 872 bp (*Lepidocephalus macrochir*) to 990 bp (*C. takatsuensis*) (Supplementary Table 2) [44]. Three domains are conserved and can be recognized in Cobitinae mitogenomes (Fig. 3c). They are terminal associated sequences (TAS), the central conserved-blocks (CSB-D, CSB-E and CSB-F) and conserved sequence blocks (CSB-1, CSB − 2 and CSB-3).

### Usage bias of start and stop codon, codon distributions and relative synonymous codons in Cobitinae mitogenomes

**Fig. 4:**
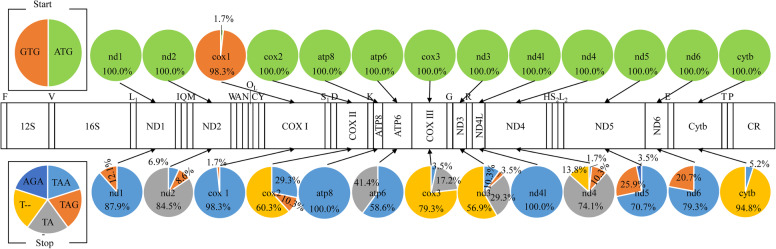
Usage bias of start and stop codons of 13 PCGs in Cobitinae mitogenomes. Pie graphs show the use frequency of start and stop codons. Gene abbreviations are the same as Table 2

The typical start codon ATG is conservative and is used in 12 PCGs, while GTG is only used in *cox1* in 98% (57/58) analyzed Cobitinae mitogenomes except one individual of *M. anguillicaudatus* (No. 11) (Fig. 4, Supplementary Table 3). Five types of stop codons were found, containing three canonical (TAA, TAG and AGA) and two truncated stop codons (TA- and T--) (Fig. 4). The two truncated termination codons are used in *nd2*, *cox2*, *atp6*, *cox3*, *nd3*, *nd4* and *cytb*, the 3′ -ends of which are followed by a tRNA gene encoded with the same strand.

**Fig. 5:**
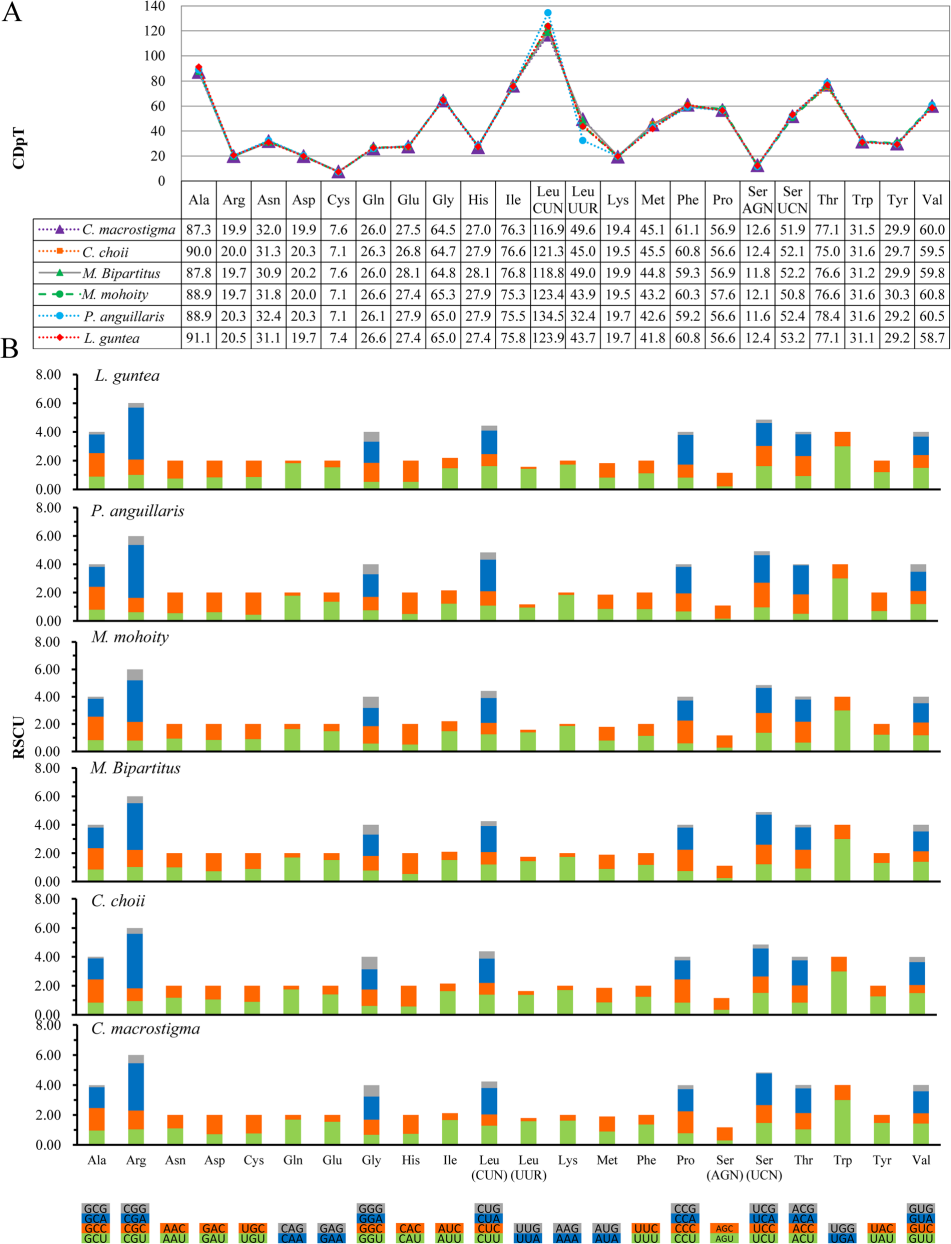
Codon distribution (**a**) and relative synonymous codon usage (**b**) of PCGs in *C. macrostigma* and other five representative species of Cobitinae. CDpT = codons per thousand codons

The codon distribution and relative synonymous codon usage (RSCU) of 58 Cobitinae mitogenomes were analyzed. Our results show that codon distribution is largely coincident among these Cobitinae mitogenomes (Supplementary Figure S1). As shown by six representative species of Cobitinae, the codons encoding Leu^(CUN)^, Ala and Thr are the three most frequently present, while those encoding *Cys* are rare (Fig. 5a). Compared to the other five Cobitinae species, *P. anguillaris* uses more codons of *Leu*^*(CUN)*^ and less codons of *Leu*^*(UUR)*^. The patterns of RSCU are also consistent among the analyzed species (Fig. 5b). Degenerated codons are biased to use more A/T than G/C in the 3rd position of PCGs, which results in the content of A + T is higher than G + C in the 3rd position of Cobitinae PCGs. For example, the codons for Arginine CCA and the codes for Tryptophan UGU are prevalent, while their other synonymous codons are relatively less used.

### A + T %, AT-skew and their linear correlations of Cobitinae mitogenomes

**Fig. 6:**
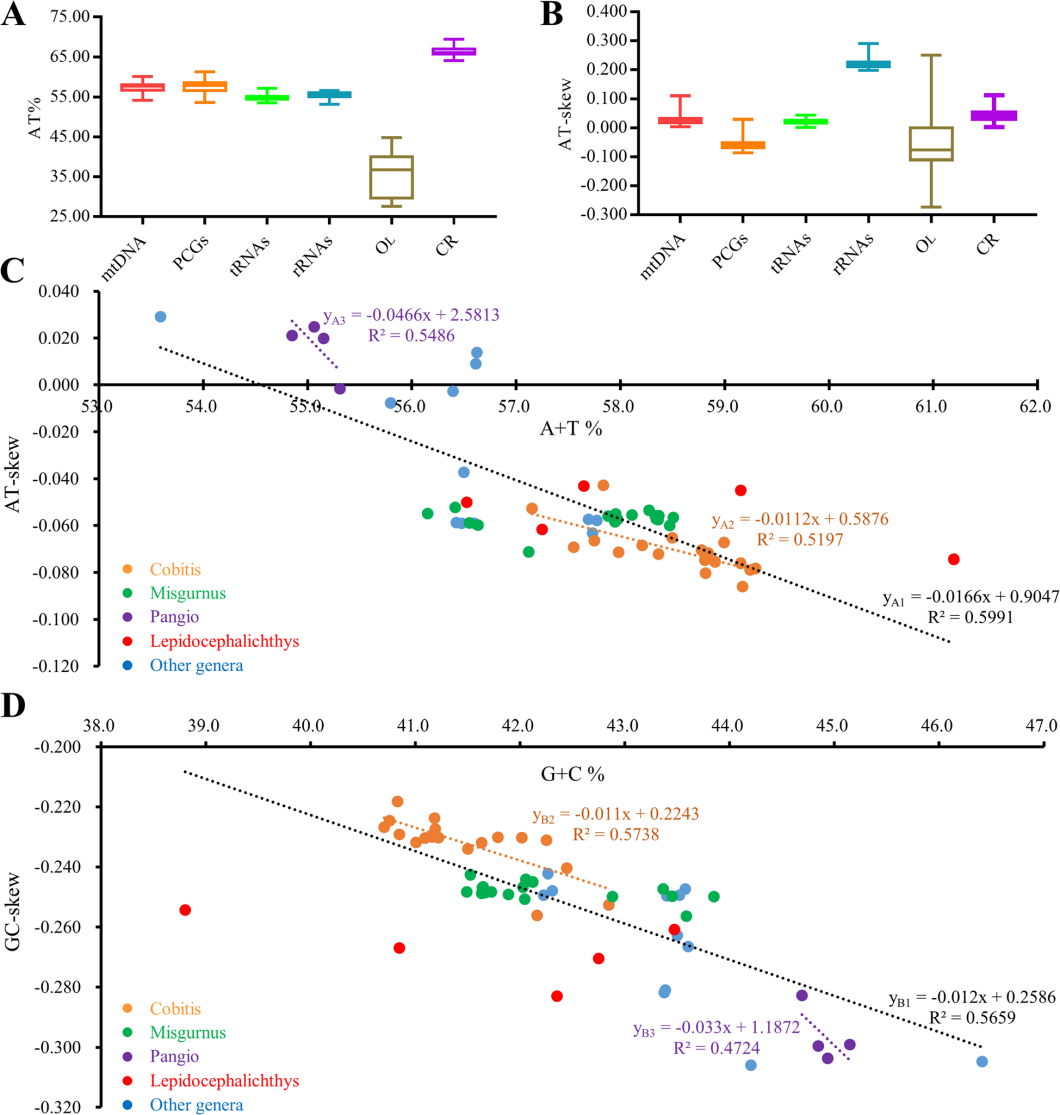
Base compositions and AT-skew in Cobitinae mitogenomes. **a**. A + T content of different regions in Cobitinae mitogenomes. **b**. AT-skew of different regions in Cobitinae mitogenomes. **c**. The correlations between A + T% and AT-skew in 13 PCGs of Cobitinae mitogenomes. **d**. The correlations between G + C% and GC-skew in 13 PCGs of Cobitinae mitogenomes

The A + T content and AT-skew of whole mitogenomes, PCGs, tRNAs, rRNAs and CR were calculated (Fig. 6a-b). The 58 Cobitinae mitogenomes all exhibit AT bias, and the A + T content is the lowest (54.8 ± 0.6%) in tRNAs and the highest (66.3 ± 0.9%) in CR (Fig. 6a, Supplementary Table 2). The AT-skew values are the largest and positive in rRNAs, while they are the smallest in PCGs and most are negative except *Canthophrys gongota*, *Acantopsis choirorhynchos*, *P. cuneovirgata*, *P. kuhlii*, *P. oblonga*, and *Kottelatlimia pristes* (Fig. 6, Supplementary Table 2). These results indicate that PCGs are biased towards using T not A in most Cobitinae mitogenomes. To examine whether the A + T content and AT-skew are different in three codon position of PCGs, we also selected the six Cobitinae species for a more detailed analysis. The A + T content shows 1st < 2nd <3rd in the three position of PCGs in all analyzed fishes. Meanwhile, the AT-skew of 1st and 3rd are positive while 2nd is negative (Table 3). This is due to the bias usage of relative synonymous codons (Fig. 5b). In all analyzed Cobitinae mitogenomes, CRs possess more A and C with all AT-skew values positive (0.002–0.112) and GC-skew negative (− 0.341−− 0.101) (Supplementary Table 2).

**Table 3:** Base composition and skewness of the mitogenomes in *C. macrostigma* and other five representative species of Cobitinae

	Size (bp)	A%	T (U)%	C%	G%	AT%	GC%	AT-skew	GC-skew		Size (bp)	A%	T (U)%	C%	G%	AT%	GC%	AT-skew	GC-skew
*C. macrostigma*										*Canthophrys gongota*									
all mtDNA	16,636	29.5	28.8	25.1	16.6	58.3	41.7	0.013	−0.205	all mtDNA	16,561	31.1	25.6	27.3	16.0	56.7	43.3	0.096	− 0.260
PCGs	11,472	27.3	31.5	25.4	15.9	58.8	41.2	−0.038	− 0.266	PCGs	11,425	28.6	28.1	27.8	15.6	56.6	43.4	0.009	− 0.281
1st of PCGs	3814	26.2	23.2	24.7	25.9	49.4	50.6	0.063	0.025	1st of PCGs	3798	29.1	20.2	27.0	23.7	49.3	50.7	0.179	− 0.064
2nd of PCGs	3814	18.2	40.9	27.3	13.6	59.1	40.9	−0.385	−0.335	2nd of PCGs	3798	19.5	38.9	27.8	13.8	58.4	41.6	−0.331	− 0.337
3rd of PCGs	3814	37.3	30.2	24.3	8.2	67.5	32.5	0.105	−0.496	3rd of PCGs	3798	40.9	20.9	31.4	6.8	61.8	38.2	0.323	−0.644
tRNAs	1557	28.3	26.9	21.6	23.2	55.2	44.8	0.024	0.037	tRNAs	1558	28.6	26.2	22.3	22.9	54.8	45.2	0.044	0.014
rRNAs	2627	33.2	22.1	23.0	21.7	55.3	44.7	0.201	−0.028	rRNAs	2628	35.0	19.3	25.0	20.7	54.3	45.7	0.290	−0.095
CR	917	34.4	32.0	19.4	14.3	66.3	33.7	0.036	−0.152	CR	901	35.6	32.7	18.9	12.8	68.4	31.6	0.042	−0.193
*M. bipartitus*										*Paramisgurnus dabryanus (1)*									
all mtDNA	16,636	29.8	28.0	25.9	16.4	57.7	42.3	0.032	−0.226	all mtDNA	16,570	29.2	27.4	26.5	17.0	56.6	43.4	0.031	−0.219
PCGs	11,471	27.4	30.6	26.3	15.8	58.0	42.0	−0.055	−0.251	PCGs	11,433	26.6	29.9	27.2	16.4	56.4	43.6	−0.059	− 0.247
1st of PCGs	3814	26.1	22.9	24.9	26.1	49.0	51.0	0.065	0.022	1st of PCGs	3800	26.7	21.5	26.8	25.1	48.2	51.8	0.107	−0.032
2nd of PCGs	3814	18.3	40.8	27.3	13.5	59.2	40.8	−0.381	− 0.339	2nd of PCGs	3800	19.7	39.2	27.4	13.6	58.9	41.1	−0.332	−0.336
3rd of PCGs	3814	37.6	27.8	26.8	7.8	65.4	34.6	0.149	−0.550	3rd of PCGs	3800	36.2	25.7	29.7	8.4	61.9	38.1	0.170	−0.557
tRNAs	1563	28.3	26.7	21.7	23.2	55.1	44.9	0.029	0.034	tRNAs	1559	28.3	26.5	22.1	23.1	54.8	45.2	0.033	0.021
rRNAs	2628	34.1	21.9	22.9	21.1	56.0	44.0	0.219	−0.042	rRNAs	2631	33.8	21.2	23.7	21.3	55.0	45.0	0.229	−0.052
CR	916	34.3	31.2	19.9	14.6	65.5	34.5	0.047	−0.152	CR	913	36.0	31.3	18.4	14.2	67.4	32.6	0.070	−0.128
*P. anguillaris*										*L. guntea*									
all mtDNA	16,602	30.1	25.4	28.0	16.4	55.6	44.4	0.084	−0.261	all mtDNA	16,566	29.3	27.8	26.7	16.2	57.1	42.9	0.026	−0.244
PCGs	11,432	27.6	27.7	28.7	16.0	55.3	44.7	−0.002	− 0.283	PCGs	11,427	26.9	30.4	27.2	15.6	57.3	42.7	−0.062	−0.270
1st of PCGs	3800	26.0	21.2	26.5	26.3	47.2	52.8	0.103	−0.005	1st of PCGs	3800	25.8	22.5	25.5	26.2	48.3	51.7	0.068	0.013
2nd of PCGs	3800	18.3	40.5	27.6	13.6	58.8	41.2	−0.376	−0.342	2nd of PCGs	3800	18.1	40.5	27.8	13.6	58.6	41.4	−0.382	−0.342
3rd of PCGs	3800	38.2	21.3	32.1	8.4	59.6	40.4	0.283	−0.586	3rd of PCGs	3800	36.5	28.1	28.4	7.1	64.6	35.4	0.131	−0.602
tRNAs	1559	27.8	27.1	21.4	23.7	54.8	45.2	0.013	0.051	tRNAs	1559	27.6	27.5	21.3	23.6	55.1	44.9	0.001	0.051
rRNAs	2635	33.7	19.4	25.4	21.4	53.2	46.8	0.269	−0.084	rRNAs	2623	33.6	21.5	24.1	20.8	55.1	44.9	0.221	−0.075
CR	928	35.0	32.0	19.7	13.3	67.0	33.0	0.045	−0.196	CR	920	32.5	31.5	21.4	14.6	64.0	36.0	0.015	−0.190

The correlations of Cobitinae mitogenomes (y_A1_ = − 0.0166x – 0.9047, R^2^ = 0.5991) genus *Cobits* (y_A2_ = − 0.012x + 0.5786, R^2^ = 0.5197) and *Pangio* (y_A3_ = = − 0.0466x + 2.5813, R^2^ = 0.5486) were calculated between A + T % versus AT-skew. All of them showed negative linear correlations, implying that AT-skew becomes more positive with the increasing of A + T content (Fig. 6c). The similar negative linear correlations were also found in G + C % versus GC-skew (Fig. 6d).

### Non-synonymous and synonymous substitutions

**Fig. 7:**
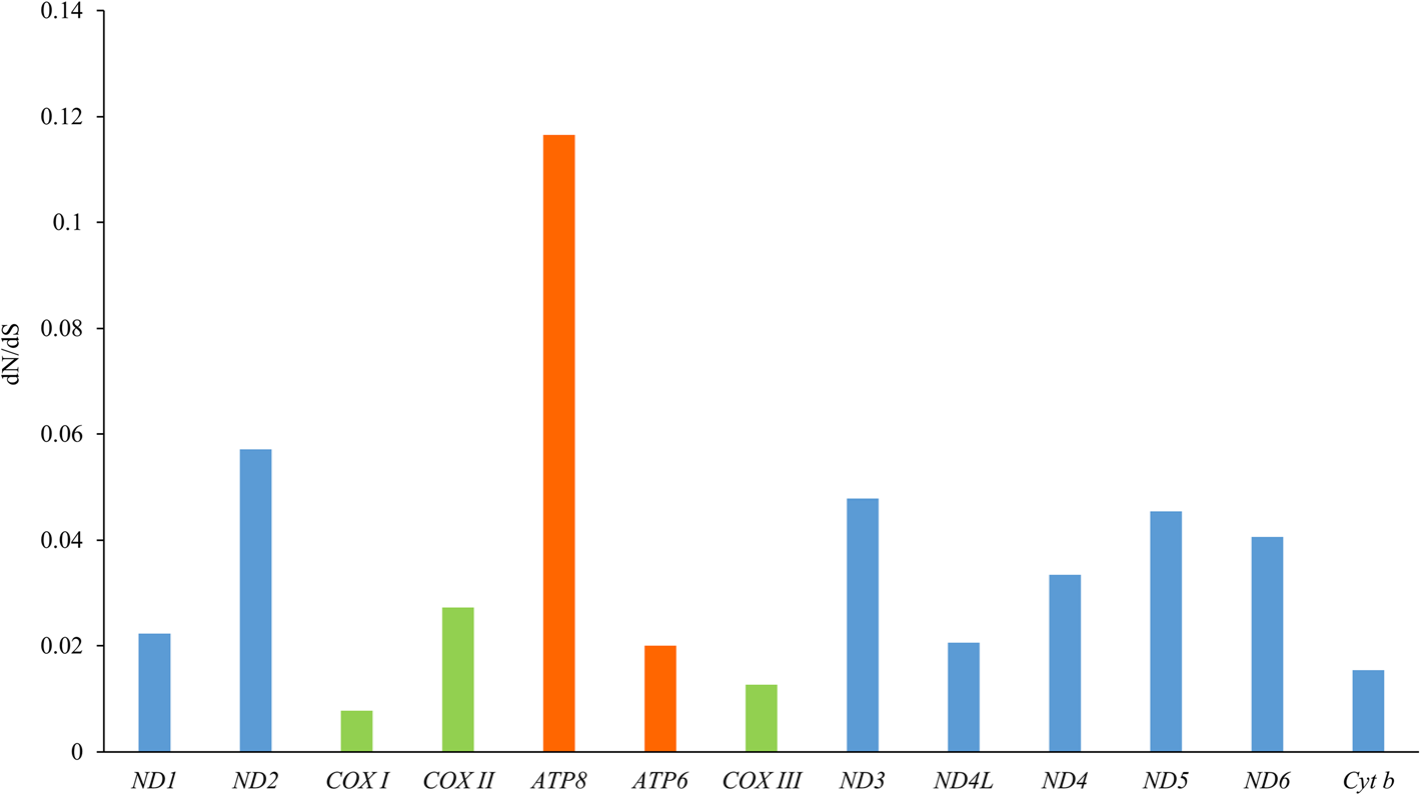
Nonsynonymous/synonymous ratios (ω = dN/dS) of the 13 PCGs of Cobitinae mitogenomes

To better understand the role of selective pressure and evolutionary relations of Cobitinae fishes, the ω or dN/dS value of each PCG was calculated (Fig. 7). All the PCGs evolved under a purifying selection (ω < 0.5). The *atp8* gene showed the highest ω value (ω = 0.12) and the *cox* family genes were lowest (ω = 0.02 ± 0.01). This phenomenon is also found in most Metazoa [56], but the fold change (> 10 fold) is particularly high in Cobitinae. The lower ω value represents less variations in amino acids. Thus, *cox1*, *cox3* and *cytb* are potential barcoding markers for Cobitinae species identification.

### Phylogenetic analysis of Cobitinae fishes

**Fig. 8:**
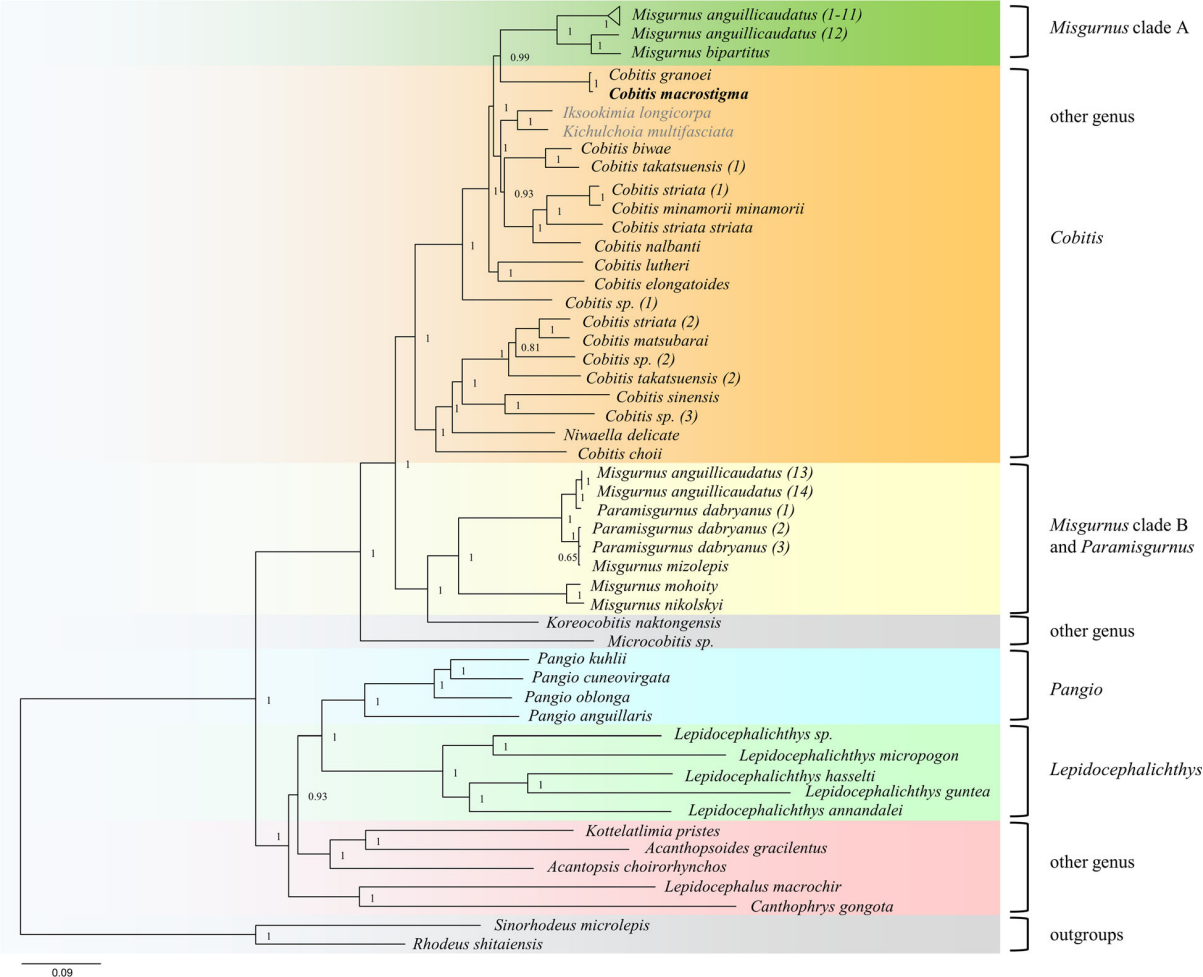
Phylogenetic tree constructed by BI methods, based on 13 PCGs of 58 Cobitinae mitogenomes. *Sinorhodeus microlepis* and *Rhodeus shitaiensis* were chosen as outgroups. Node numbers represent the values of posterior probability

Molecular phylogenetic analyses were performed using 13 PCGs from 58 Cobitinae mitogenomes, belonging to 41 species from 14 genera. The ML and BI analyses generated similar topology with high bootstrap support / posterior probability values. Each tree was similarly divided into two main clades: *Cobitis*-*Misgurnus*-other genera (clade I) and *Pangio*-*Lepidocephalichthys*-other genera (clade II) (Fig. 8 and Supplementary Figure S2). Clade I included all analyzed species of *Cobitis*, *Paramisgurnus* and *Misgurnus*, and five species from other genus (*I. longicorpa*, *K. multifasciata*, *N. delicata*, *K. naktongensis*, and *Microcobitis sp.*). Four *Pangio* species, five *Lepidocephalichthys* species and other five species (*K. pristes*, *A. choirorhynchos*, *A. gracilentus*, *L. macrochir*, and *C. gongota*) were clustered into Clade II, among which the analyzed species of genus *Pangio* and *Lepidocephalichthys* formed two well-supported (pp = 1.00) monophyletic groups respectively. In addition, *Pangio* is the sister genus to *Lepidocephalichthys*.

The BI phylogenetic tree confirmed that *Cobitis* was a paraphyletic group, since *Misgurnus* clade A, *N. delicate*, *I. longicorpa*, and *K. multifasciata* shared the common ancestor with the all 15 *Cobitis* species analyzed in this study, with high posterior probability values (pp = 1.00). The species of *Misgurnus* were separated into two independent lineages: the majority of *M. anguillicaudatus* individuals (12/14) and *M. bipartitus* clustering with the *Cobitis* species (*Misgurnus* clade A), and two *M. anguillicaudatus* individuals, *M. mizolepis*, *M. mohoity*, and *M. nikolskyi* gathering with *P. dabryanus* and *K. naktongensis* (*Misgurnus* clade B).

### Divergence time estimation of Cobitinae fishes

**Fig. 9:**
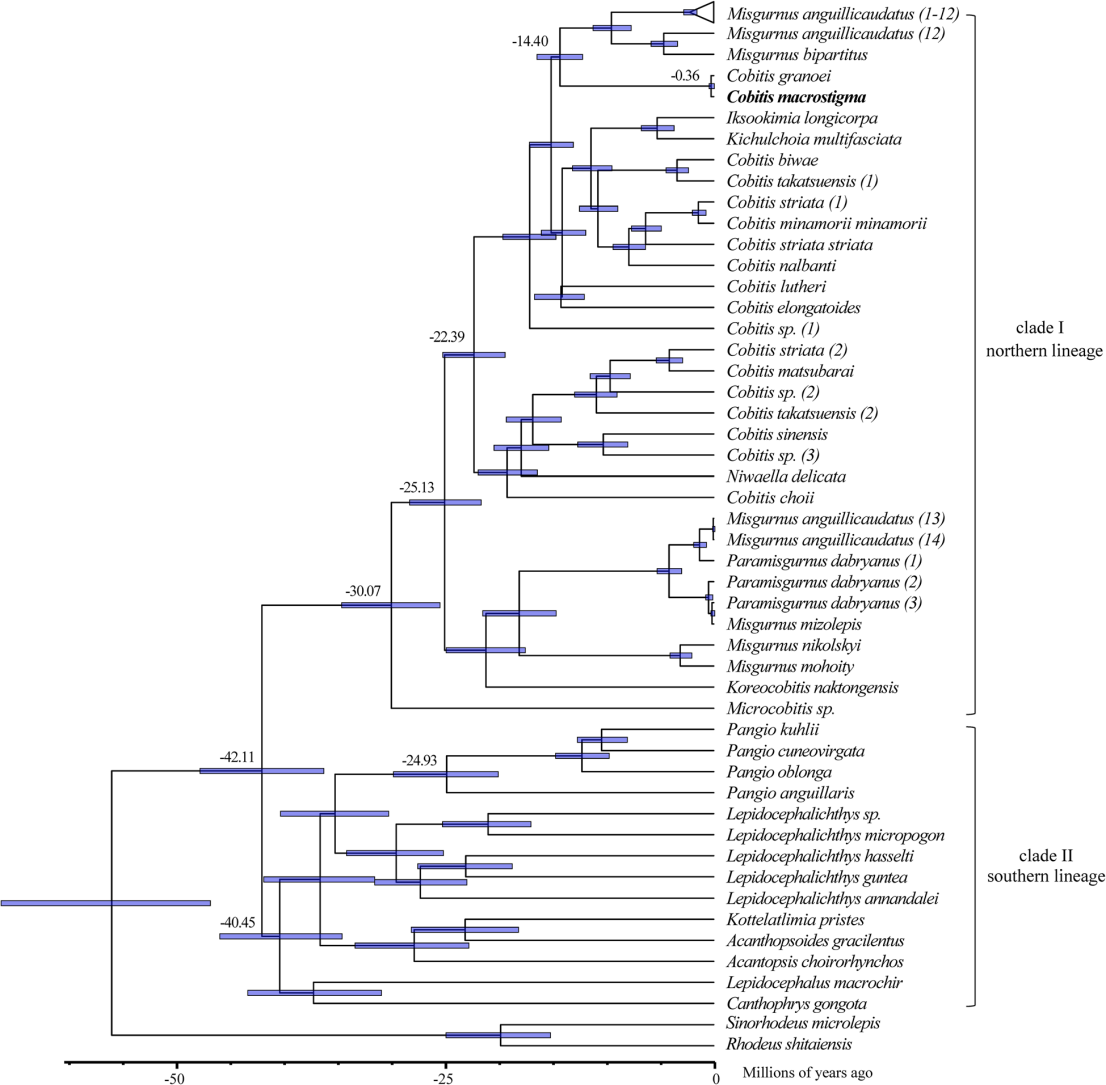
The divergence times of Cobitinae fishes. The ranges of 95% HPD intervals are represented by the blue bars

The combination of strict clock model and Yule process tree prior provided the best fit to the data sets (Supplementary Table 4). The chronogram with divergence time of Cobitinae lineages was estimated based on the cytB mutation rate (0.68% per million years) (Fig. 9). The first split of Cobitinae lineages was estimated to have occurred in the late Eocene (42.11 Ma, 95% HPD: 36.35–47.86 Ma), then separated into clade I (northern clade) and clade II (southern lineages). *Cobitis*-*Iksookimia*-*Kichulchoia*-*Niwaella* lineage diverged from the rest of northern clade lineage during the Oligocene (30.07 Ma, 95% HPD: 25.55–34.69 Ma), similar to the previous described [35], then diversified and further radiated after 4.94 Ma. The mtDNA introgression between ancestral species of *Cobitis* and ancestral species of *Misgurnus* seems to have taken place in the Middle Miocene (14.40 Ma, 95% HPD: 12.30–16.54 Ma). *C. macrostigma* appeared about 0.36 Ma (95% HPD: 0.06–0.55 Ma) in the Pleistocene. *Pangio*-*Lepidocephalichthys*-other genera (southern lineages) might originate about 40.45 Ma. In southern lineages, *Pangio* was estimated to have occurred about 20.14–29.88 Ma, and the divergence times of the four species analyzed in this study are congruent with the previous described dating [24].

## Discussion

In this study, we conducted a comparative mitogenome analysis and revealed the conserved and unique characteristics of 58 Cobitinae mitogenomes. Cobitinae mitogenomes display highly conserved tRNA secondary structure, overlaps and non-coding intergenic spacers. Among the 22 tRNAs, *tRNA*^*ser(AGN)*^ (*S1*) is the only one that is not folded into the typical clover-leaf secondary structure (Fig. 2a). Loss of stem in *S1* is common character among Cobitinae and other metazoan mitogenomes [57, 58]. Similarly, the widespread unmatched base pairs among Cobitinae tRNAs is also a conserved feature in the eukaryote mitogenome [59–61]. Although their functions are not clear in fish, the unmatched base pairs are considered as the current state of evolutionary and irreversible process, which might be caused by tRNA editing [62].

Like other cyprinid fishes [14, 63], two long overlaps and two long IGSs were found in Cobitinae mitogenomes. The motif “ATGCTAA” in *nd4l*-*nd4* was conserved in vertebrates, including fish, turtle and human [14, 63–66]. However, in comparison with the conserved motif (ATGATAA) in other Cypriniformes fishes, there is a specific 3 bp insertion (GCA) in the *atp8*-*atp6* overlap motif of Cobitinae and other loaches [67–69], indicating this insertion is a characteristic feature of loaches. IGSs are important for transcription and associated with gene rearrangement in insects [70–72]. It is commonly assumed that IGS had a rapid nucleotide substitution rate under relaxed selection [73]. Moreover, Cobitinae mitogenomes share highly conserved sequences in IGSs that are immediately adjacent to tRNAs, such as “CTTTCCCGCC”, “AAGGCGGGA” and “AGC”. Whether these conserved sequences have a function or not and how they act awaits further investigation. As the longest IGSs, CR plays an important role in controlling the transcription and replication of mtDNA molecule by several domains and motifs [74, 75]. Although significant length variation were found in CR of vertebrate [76], the three domains can also be recognized in Cobitinae mitogenomes. Furthermore, the AT-skew and GC-skew of CR might reflect the strand asymmetry [77–79]. In teleost, the skew inversion of CR was only found in the mitogenomes of *Albula glossodonta* and *Bathygadus antrode*, showing a reversed strand asymmetry [75]. The normal Cobitinae mitogenomes CR skewness indicates that the strand asymmetry is not reversed.

The phylogenetic analyses show the monophyly of the genus *Pangio* and *Lepidocephalichthys*, consistent with the previous study [35]. However, *Cobitis*, the biggest genus of Cobitinae [15], is a complex and controversial paraphyletic group. Similar to the trees constructed by *cyt b* [25, 80, 81], *Iksookimia*, *Kichulchoia* and *Niwaella* species were nested within *Cobitis*, implying a close relationship among them. Perdices [37] proposed that these species of *Iksookimia*, *Kichulchoia*, and *Niwaella* might belong to genus *Cobitis*, as morphologically specialized species derived from a local *Cobitis* species. However, this assumption awaits more morphological, karyological and molecular investigation. In addition, our phylogenetic analysis confirmed the assumption that *M. mizolepis* and *P. dabryanus* are conspecific [33, 80] and the different lineages under the species name *C. striata* and *C. takatsuensis* might actually represent different species.

The species of *Misgurnus* were separated into two independent clade and clustered into *Cobitis* species and *P. dabryanus-K. naktongensis*, respectively. The same results were observed in the trees based on the *cyt b* [80] and 13 PCGs from 28 cobitidae species [47]. However, all *Misgurnus* and *Koreocobitis* species were grouped into a monophyletic clade when their phylogenetic relationships were constructed by nuclear gene *rag-1* [80]. This incongruity between mitochondrial and nuclear gene trees was explained by the different evolutionary rate of markers, hybridization or introgression [82]. It is commonly believed that hybridization and subsequent mtDNA introgression might occur between ancestral species of *Cobitis* and ancestral species of *Misgurnus* [35, 80]. In this study, we collected 14 mitogenomes from *M. anguillicaudatus*, which were divided into two genetically divergent clades. The similar phenomenon has been reported by several previous studies, which is explained by hybridization and mtDNA introgression [34, 35, 47, 83, 84]. Considering that *M. anguillicaudatus* clustered into the clade of *Misgurnus* and *Koreocobitis* by nuclear analyses [80], we supposed that the 12 mitogenomes (No. 1–12) of *M. anguillicaudatus* in *Misgurnus* clade A could be considered as the introgressed mtDNA type because of their close relationship with *Cobitis* species, whereas the other two individuals in *Misgurnus* clade B retained the original *M. anguillicaudatus* mitogenomes. *M. anguillicaudatus* with introgressed mtDNA type spread over most of East Asia, including China, Japan and Korea. *M. anguillicaudatus* shows extensive ploidy variability in nature. Besides most common diploid individuals (2n = 50), triploid (3n = 75) and tetraploid (4n =100) have been frequently recorded in some localities of China and Japan [21, 47, 85, 86]. Rare pentaploid (5n = 125) and even hexaploid (6n = 150) individuals were found in the Yangtze River basin [87]. All of *M. anguillicaudatus* polyploids analyzed in this study belonged to the introgressed mtDNA type. Since mtDNA is inherited maternally, these polyploids might have originated from the diploid *M. anguillicaudatus* with introgressed mtDNA. Further analyses are needed to confirm this hypothesis of inter-genus mtDNA introgression based on a large-scale sampling with quantitative morphological features, definite ploidy, and more genes from both mitochondria and nuclear genomes.

The first split of Cobitinae lineages was estimated to have occurred in the late Eocene (42.11 Ma, 95% HPD: 36.35–47.86 Ma), separating northern clade and southern lineages, consistent with reconstruction dates of the paleo-drainages of East Asia [35, 88]. Cobitinae fishes in Clade I and Clade II, nominated as “northern clade” and “southern lineages” respectively, show a distinct disjunctive distribution with a small area of sympatry in Vietnam [35]. Consistent with their locations, the northern clade spread to most of East Asia, Siberia and Europe, while the southern lineages distribute across the Indian subcontinent and Southeast Asia after their isolation. The nodes within northern clade and southern lineage appear asynchronous, implying that some local dominant factors, rather than large-scale events, might shape the evolution within northern or southern lineage.

## Conclusions

This study represents the first comparative mitogenome and phylogenetic analyses within Cobitinae. The conserved and unique characteristics of 58 Cobitinae mitogenomes were revealed. We observed distinct base compositions among different genus and identified a specific 3 bp insertion (GCA) in the *atp8*-*atp6* overlap as a unique feature of loaches. ML and BI analyses both strongly support the paraphyly of *Cobitis* and polyphyly of *Misgurnus*. In addtion, Cobitinae might have split into northern and southern lineages in the late Eocene (42.11 Ma), and a mtDNA introgression between *Cobitis* and *Misgurnus* might have occured about 14.40 Ma. The current study provides new insights into the mitogenome features and evolution of Cobitinae fishes.

## Methods

### Sampling, sequencing and assembly

The *C. macrostigma* analyzed in this study was caught from the Yangtze River in Yibin City, Sichuan Province, China (N: 28°46′6.01″, E: 104°38′13.99″) in October 2018 and five individual were transported to the laboratory (National Aquatic Biological Resource Center, NABRC) in oxygen-rich water. It possesses 5–9 large and round spot in the midline of lateral body side [89] (Fig. 1). Before sampling, they were reared in a square and glass recirculating freshwater tanks with a volume of about 100 L, at 22 °C on a 14 h (hour) light/10 h dark cycle for morphological identification. After deep and overdosed anesthesia with styrylpyridine (a common anaesthetic used in fish, 30-50 mg/L; aladdin, China), one healthy one-year-old female fish, 7 cm in length and 1.8 g in weight, was euthanized by immediately cutting off the spinal cord adjacent to the head. Total DNA was extracted according to the Ezup Column Animal Genomic DNA Kit technical manual (Sangon, Shanghai, China). PCR primers were designed based on the conserved sequences between the mitogenomes of *C. granoei* (GenBank: NC_023473.1) and *C. sinensis* (GenBank: NC_007229.1). 742–2495 bp DNA were amplified by using High Fidelity DNA Polymerase (Yeasen, Shanghai) (Supplementary Table 1). To obtain accurate sequences, we chose a cloning strategy. According to manual, PCR amplicon was purified, ligated ESI-Blunt vector (Yeasen, Shanghai) and transfected into 5α Chemically Competent Cell (Tsingke Biological Technology, Beijing). The positive clones were sequenced by Quintara Biosciences (Wuhan, China). The segments, longer than 1500 bp, were sequenced using the primer walking sequencing strategy. The resulting DNA sequences were assembled using DNAStar (DNASTAR Inc., USA) [90]. Other 57 Cobitinae mitogenomes were download from NCBI GenBank database [38–55].

### Gene annotation and bioinformatic analyses

tRNA genes and their secondary structures were predicted with MITOS [91] and tRNAscan-SE 2.0 with default parameters [92]. All 13 PCGs and two rRNA genes were annotated by comparison with the sequences of other Cobitinae fishes in GenBank (https://blast.ncbi.nlm.nih.gov/). The mtDNA maps were drawn using CGView Server V1.0 [93]. The sequence logos of gene overlaps and non-coding IGSs were drawn using WebLogo 3.7.4 [94]. The base composition, codon distributions and relative synonymous codons usage were calculated using DNAStar (DNASTAR Inc., USA) [90], MEGA 7.0 [95] and Microsoft Excel 2010. Skewness was measured using the formulas: AT-skew = (A% - T%) / (A% + T%) and GC-skew = (G% - C%) / (G% + C%) [79]. The silimlarity of the sequences was calculated in MEGA 7.0 [95] under p-distance and NCBI-BLAST (https://blast.ncbi.nlm.nih.gov/Blast.cgi).

### Phylogenetic analyses

The phylogenetic analysis was performed based on 13 PCGs of 58 Cobitinae mitogenomes. *Sinorhodeus microlepis* and *Rhodeus shitaiensis* were chosen as the outgroups (Table 1). Each of the 13 gene sequences was separately aligned using Muscle v3.8.31 [96] and concatenated into a sequence matrix by PhyloSuite v1.2.2 [97]. Then PartitionFinder2 [98] was used to find the best partitioning strategy and to calculate the best-fit evolutionary models for each subset. For the alignment, a scheme with eight partitions was selected and GTR + G + I was chosen as the best-fit evolutionary model for each partition. Phylogenetic trees were constructed by the maximum likelihood (ML) method and bayesian inference (BI). The ML method was implemented in RAxML v8.2.12 [99]. Each partition scheme was run with the GTRGAMMAI model, and 1000 rapid bootstrapping replications were set to evaluate the bootstrap support values and search for the best-scoring ML tree. The BI phylogeny was performed in MrBayes v3.1.2 [100] with the “unlink” and “prest ratepr = variable” model parameters. 10,000,000 generations were run in two independent runs of four independent Markov Chain Monte Carlo (MCMC) chains, and were sampled every 1000 generations. The convergence of the BI analyses was investigated using Tracer v1.7.1 software. The first 2500 trees were discarded as conservative burn-in, and the rests were used to generate a majority rule consensus tree.

In cobitid fishes, 0.680% (divergence per pairwise comparison per Ma) was calculated and suggested for the mutation rates of *cytb* gene [32]. In this study, BEAST v1.10.4 [101] was used to estimate the divergence time with the rate (0.68%). GTR + G + I was chosen as the best fit model by PartitionFinder2 [98]. The best-fit clock type and tree prior were selected from two clock models (strict clock and uncorrelated relaxed clock) and four tree priors (Yule process, Exponential growth, Constant size and Bayesian skyline) by comparing the marginal likelihood values estimated by path sampling [102]. The analyses were simultaneously run for 20,000,000 generations, with parameters sampled every 1000, then the first 25% of the trees were discarded as burn-in. Tracer v1.5 [103] and Figtree were used to assess the convergence and view trees, respectively.

## Supplementary Information

**Additional file 1: Table S1.** List of primers used to amplify the mitogenome of *C. macrostigma.*

**Additional file 2: Table S2.** Length, base composition and skewness of Cobitinae fish mitogenomes.

**Additional file 3: Table S3.** Start and stop codons of 13 PCGs in Cobitinae mitogenomes.

**Additional file 4: Table S4.** Marginal likelihood values of different combinations of clock model and tree prior.

**Additional file 5: Figure S1.** Codon distribution (**A**) and relative synonymous codon usage (B) of PCGs in the 58 Cobitinae mitogenomes. CDpT = codons per thousand codons.

**Additional file 6: Figure S2.** Phylogenetic tree constructed by ML methods, based on 13 PCGs of 58 Cobitinae mitogenomes. *Sinorhodeus microlepis* and *Rhodeus shitaiensis* were chosen as outgroups. Node numbers represent the bootstrap value.

## Data Availability

*C. macrostigma* mitochondrial genome has been deposited in GenBank under the accession numbers MT259034. The 59 mitogenomes from Cobitinae species, *Sinorhodeus microlepis* and *Rhodeus shitaiensis* were downloaded from GenBank. Their accession numbers and references were listed in Table 1. Other supporting results are included within the article and its additional files.

## Abbreviations

mitogenome: Mitochondrial genome
mtDNA: Mitochondrial DNA
Ma: Million years ago
tRNA: Transfer RNA
PCG: Protein coding gene
rRNA: Ribosomal RNA
O_L_: Origin of L-strand replication
CR: Control region
*cytb*: Cytochrome b
*rag-1*: Recombination activating gene 1
bp: Base pair
*nd1–6*: NADH dehydrogenase subunit 1–6
*nd4l*: NADH dehydrogenase subunit 4 L
*cox1–3*: Cytochrome oxidase subunit I-III
*atp6*: ATPase subunit 6
*atp8*: ATPase subunit 8
DHU: Dihydrouracil
IGS: Non-coding intergenic spacer
RSCU: Relative synonymous codon usage
ω or dN/dS: Non-synonymous and synonymous substitutions
HPD: Highest posterior density
ML: Maximum likelihood
BI: Bayesian inference
