# Supplementary material for: Comparative mitogenome analyses uncover mitogenome features and phylogenetic implications of the subfamily Cobitinae
Source: BMC Genomics. 2021 Jan 14;22:50. doi: 10.1186/s12864-020-07360-w (PMC7809818; doi:10.1186/s12864-020-07360-w)
Supplement: Supplementary file 1 — Additional file 1: Table S1. List of primers used to amplify the mitogenome of C. macrostigma. [file 12864_2020_7360_MOESM1_ESM.docx]

Supplementary Table 1 List of primers used to amplify the mitogenome of *C. macrostigma*

| Forward primer | Sequence (5’ → 3’) | Reverse primer | Sequence (5’ → 3’) | Tm | Position | Length/bp |
| --- | --- | --- | --- | --- | --- | --- |
| F1 | CAGCAGTGATAAATATTAAGC | R1 | CATGATGCAAAAGGTACGAGG | 52.4 | 236-1276 | 1040 |
| F2 | CAAGGGAAAGCTGAAAGAG | R2 | ATTACTCCGGTCTGAACTC | 50.1 | 1215-2586 | 1371 |
| F3 | ATTAAACCTAGCGGAAAGTGG | R3 | ACTATAGAGAGAAGTGCAGCT | 51.2 | 2273-3635 | 1362 |
| F4 | TACACTAGCAGAGACTAACCG | R4 | CCTAAAATTGATGAGACACCTG | 51.9 | 3426-5921 | 2495 |
| F5 | ATTCCTCTAATAATTGGTGC | R5 | GCTGGTTCCTCAAATGTATGG | 49.2 | 5699-6958 | 1259 |
| F6 | TACAGTCTCATCTATTGGTTC | R6 | GGGTATATTACTCATGGTAGG | 48.0 | 6805-8164 | 1359 |
| F7 | CTAACCACAGCTTTATGCC | R7 | TGTTGGCTGGTTACGCATTC | 50.0 | 7776-8518 | 742 |
| F8 | TGACCATGGCAATAAGCTTCT | R8 | TGGTGTTCAGATGTAAAGTGG | 52.4 | 8084-9334 | 1250 |
| F9 | AAGGTGAACGTAAGCAAGC | R9 | ATTAGGAAGACTTGTAAGG | 48.0 | 9308-10744 | 1436 |
| F10 | TTATGATCCACAACAACTGC | R10 | TCAGCCTCATATAAGCTCTGG | 51.2 | 10407-11691 | 1284 |
| F11 | TTTAATTACTGCTGGCTACTC | R11 | CTGTAAATAAGGTTGTAAGGG | 48.0 | 11540-12793 | 1253 |
| F12 | TAATACAGCAGCTTTACAGGC | R12 | TGCTAGTAATTTGTGCATGG | 51.2 | 12539-13942 | 1403 |
| F13 | AACATGATTTGAAGCTACAGG | R13 | TCAGATTCATTGGACTAAGGC | 49.2 | 13613-14832 | 1219 |
| F14 | TAAAGAGACCTGAAATATTGG | R14 | GGTCTAATTATTCAATAGGTG | 48.0 | 14684-15705 | 1021 |
| F15 | TTCCTAGTCCTAATCCCACTG | R15 | TCAGCTTTCGTGGAGTCAGG | 54.8 | 15441-363 | 1558 |
