# Supplementary material for: Comparative mitogenome analyses uncover mitogenome features and phylogenetic implications of the subfamily Cobitinae
Source: BMC Genomics. 2021 Jan 14;22:50. doi: 10.1186/s12864-020-07360-w (PMC7809818; doi:10.1186/s12864-020-07360-w)
Supplement: Supplementary file 4 — Additional file 4: Table S4. Marginal likelihood values of different combinations of clock model and tree prior. [file 12864_2020_7360_MOESM4_ESM.docx]

**Supplementary Table 4** Marginal likelihood values of different combinations of clock model and tree prior

| **Molecular clock model** | **Coalescent tree prior** | **Log marginal likelihood** |
| --- | --- | --- |
| **Strict clock** | **Yule process** | **-20841.462** |
| Strict clock | Exponential growth | -21007.033 |
| Strict clock | Constant size | -21263.001 |
| Strict clock | Bayesian skyline | -20911.724 |
| Uncorrelated lognormal relaxed clock | Yule process | -20953.575 |
| Uncorrelated lognormal relaxed clock | Exponential growth | -21503.364 |
| Uncorrelated lognormal relaxed clock | Constant size | -21738.838 |
| Uncorrelated lognormal relaxed clock | Bayesian skyline | -21393.133 |

The best-fitting tree prior and molecular clock model are indicated in bold font.
