# Supplementary figures and images for: Comparative mitogenome analyses uncover mitogenome features and phylogenetic implications of the subfamily Cobitinae
Source: BMC Genomics. 2021 Jan 14;22:50. doi: 10.1186/s12864-020-07360-w (PMC7809818; doi:10.1186/s12864-020-07360-w)

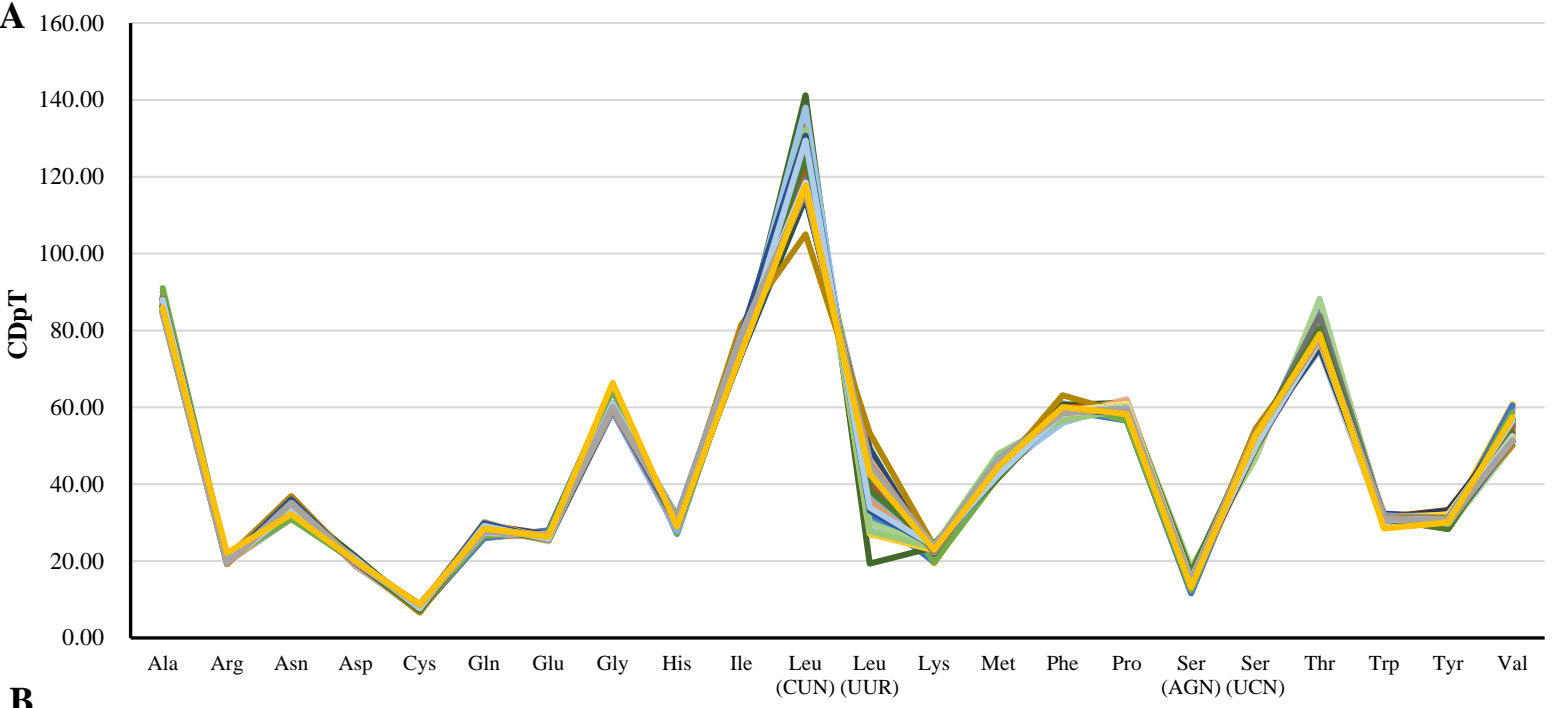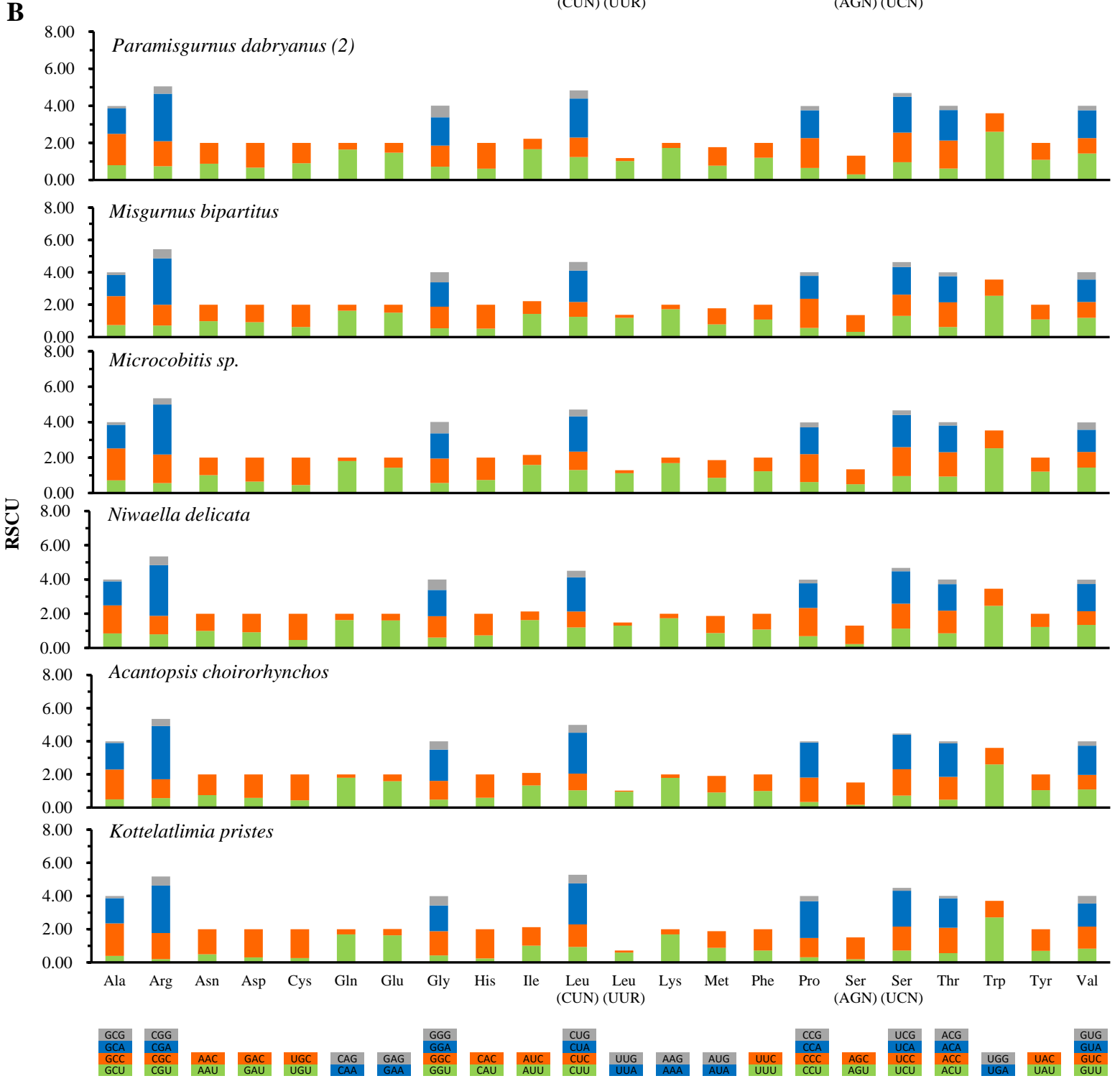

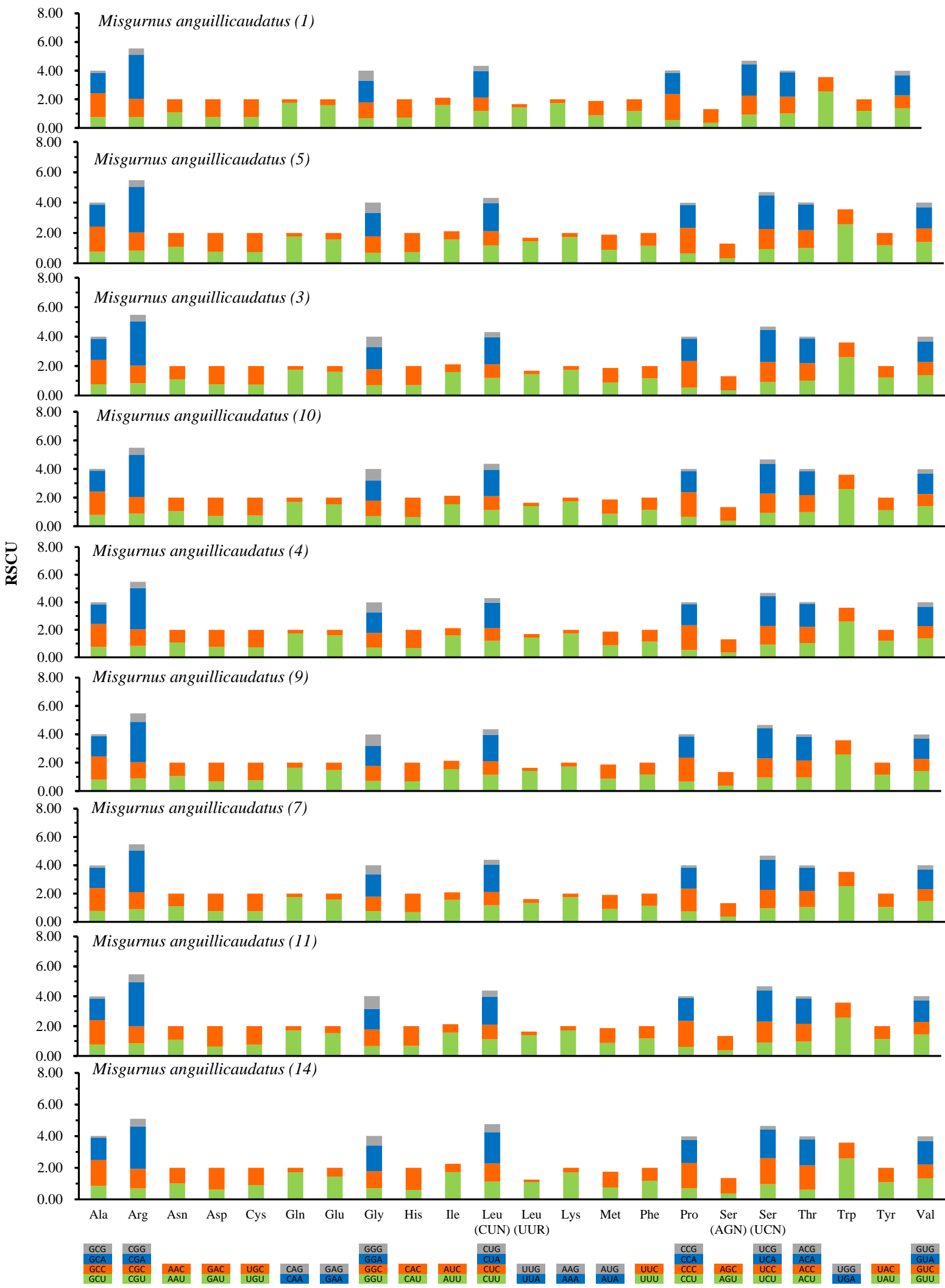

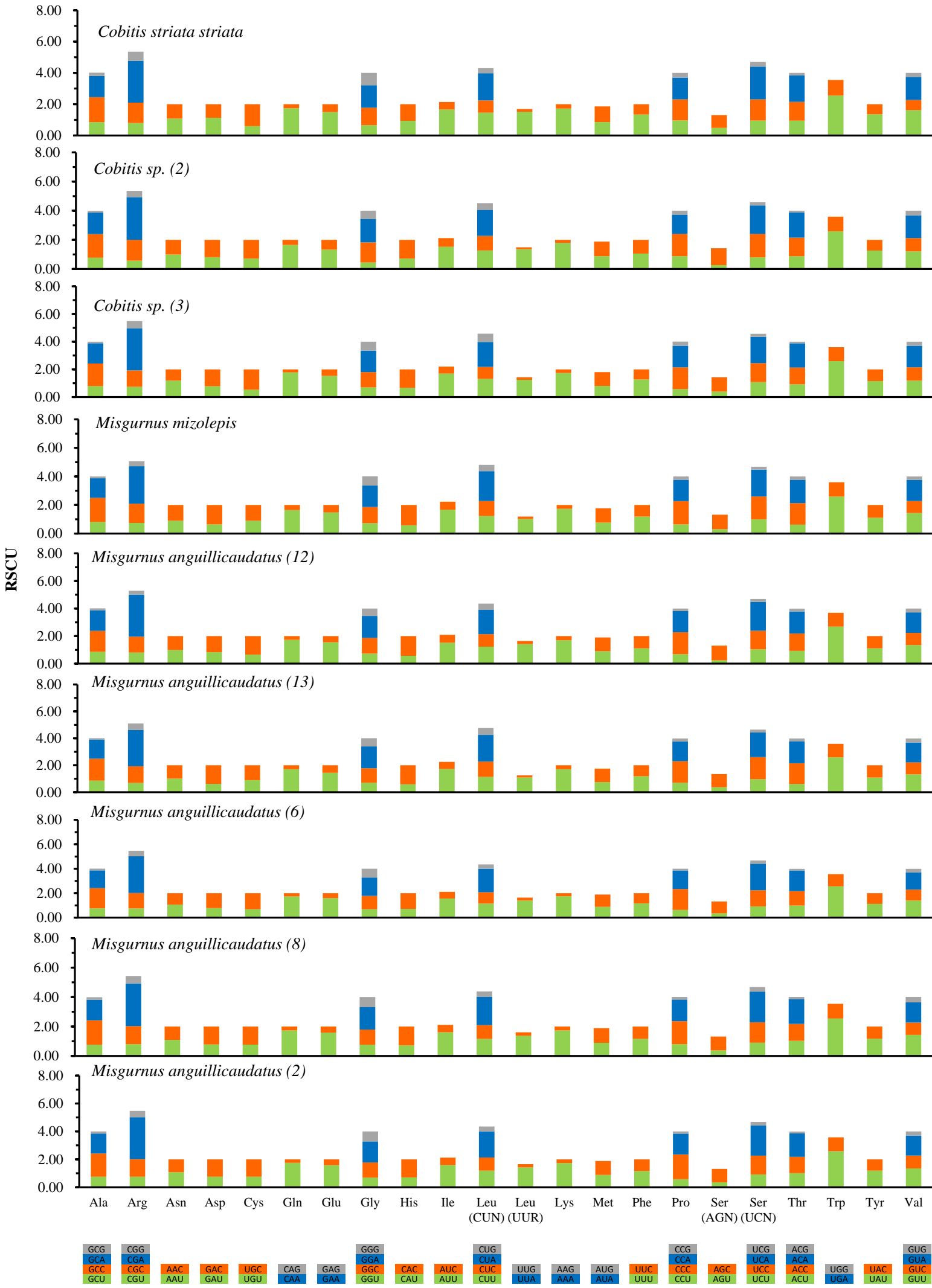

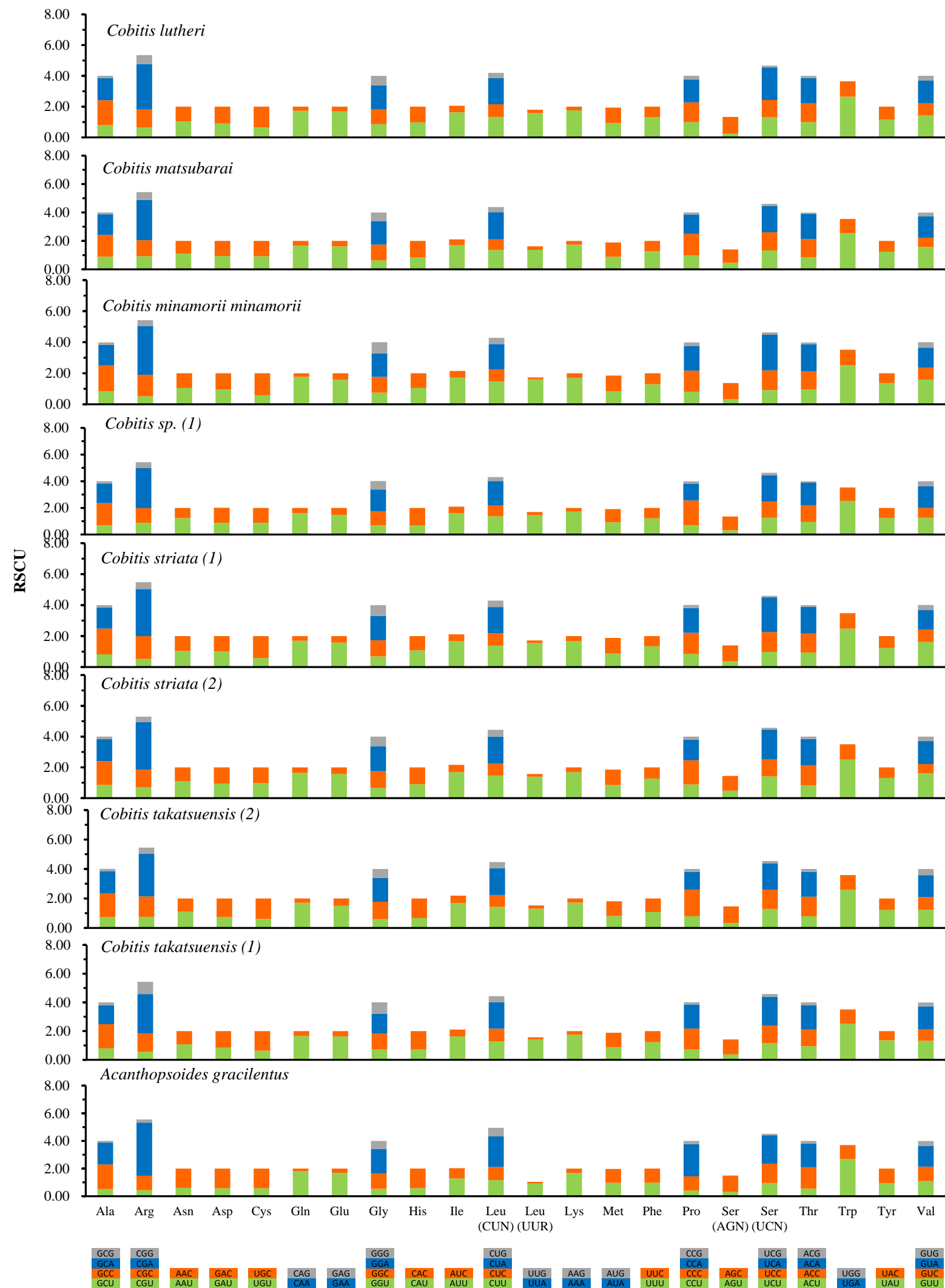

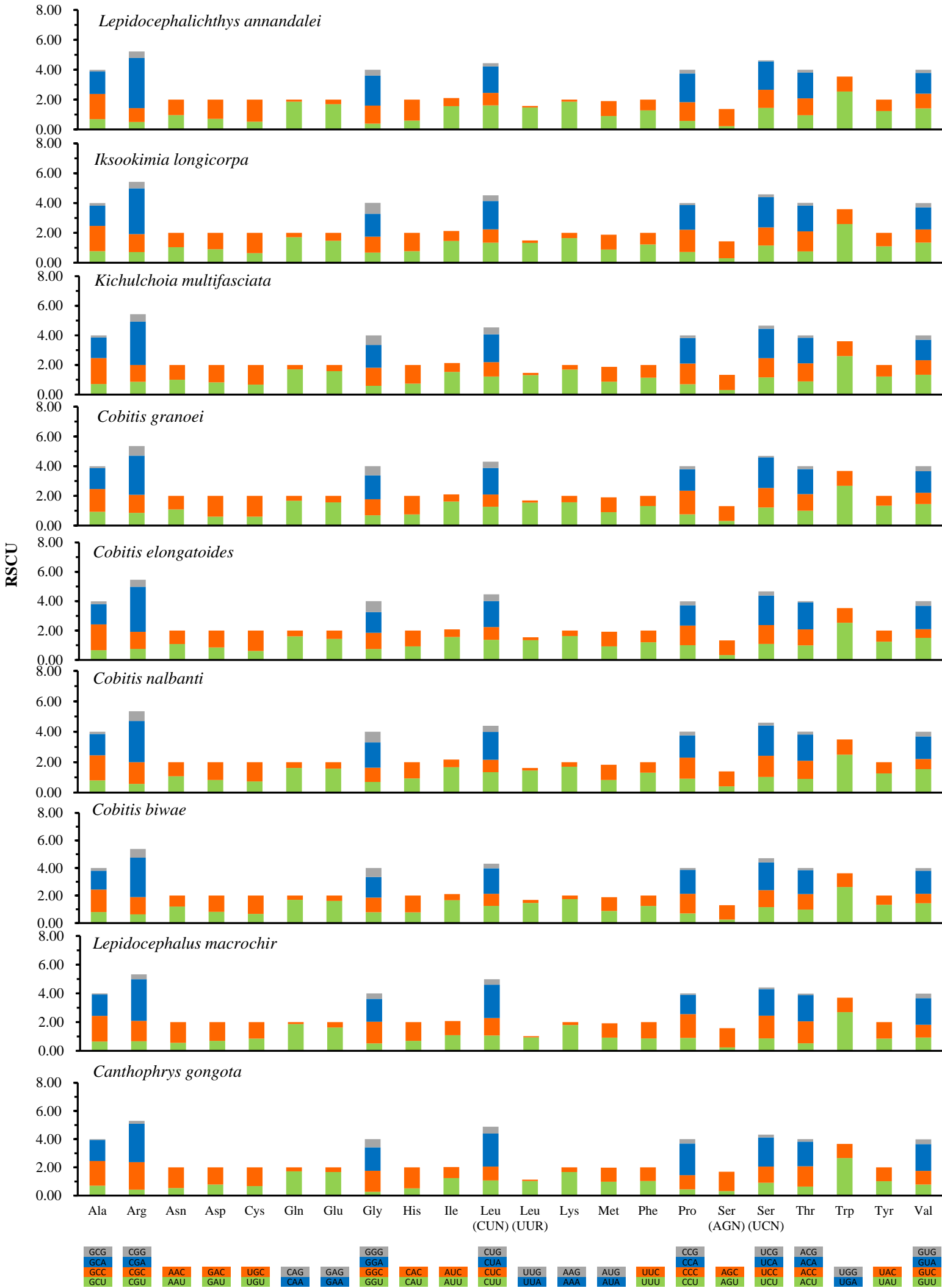

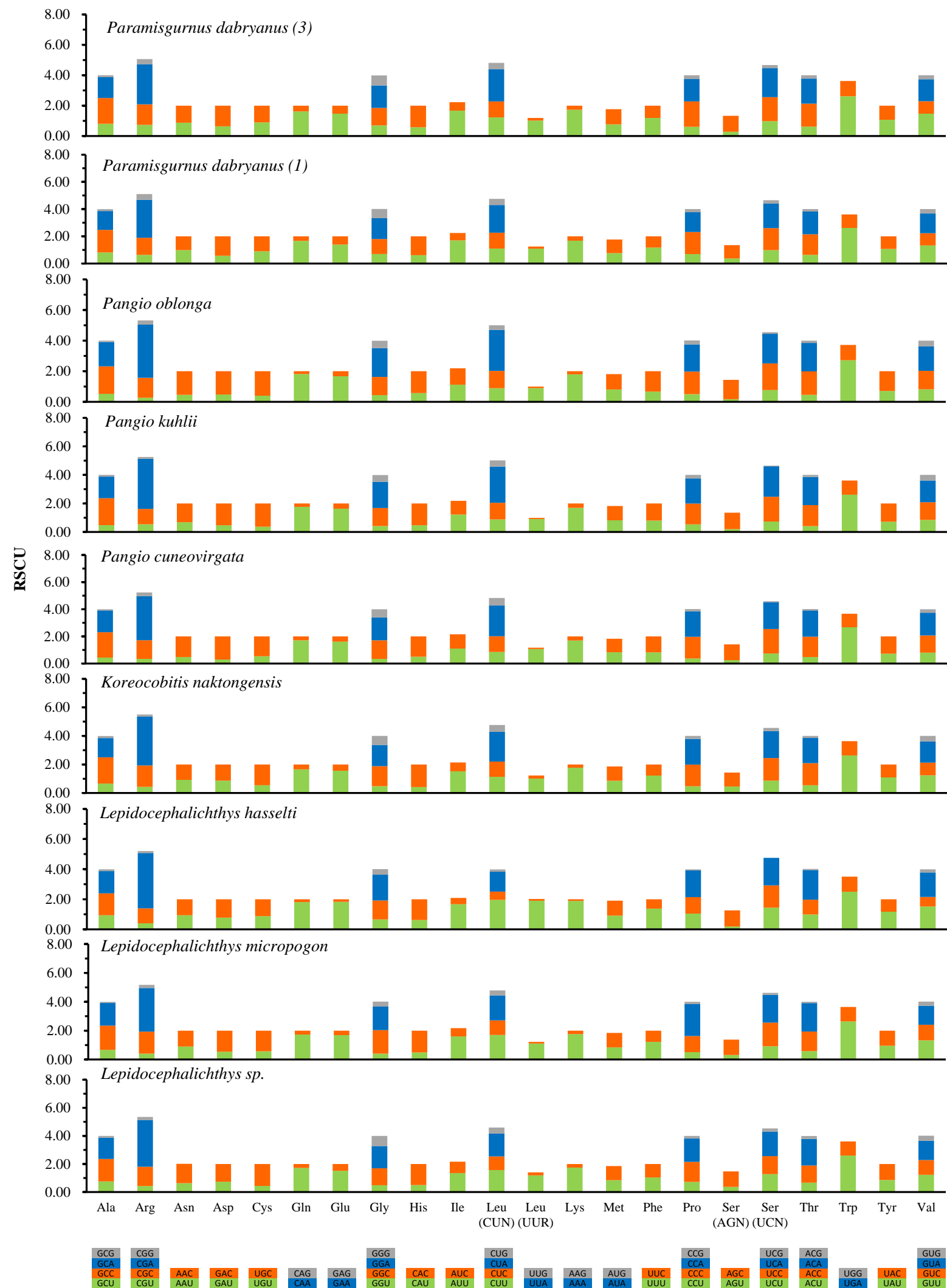

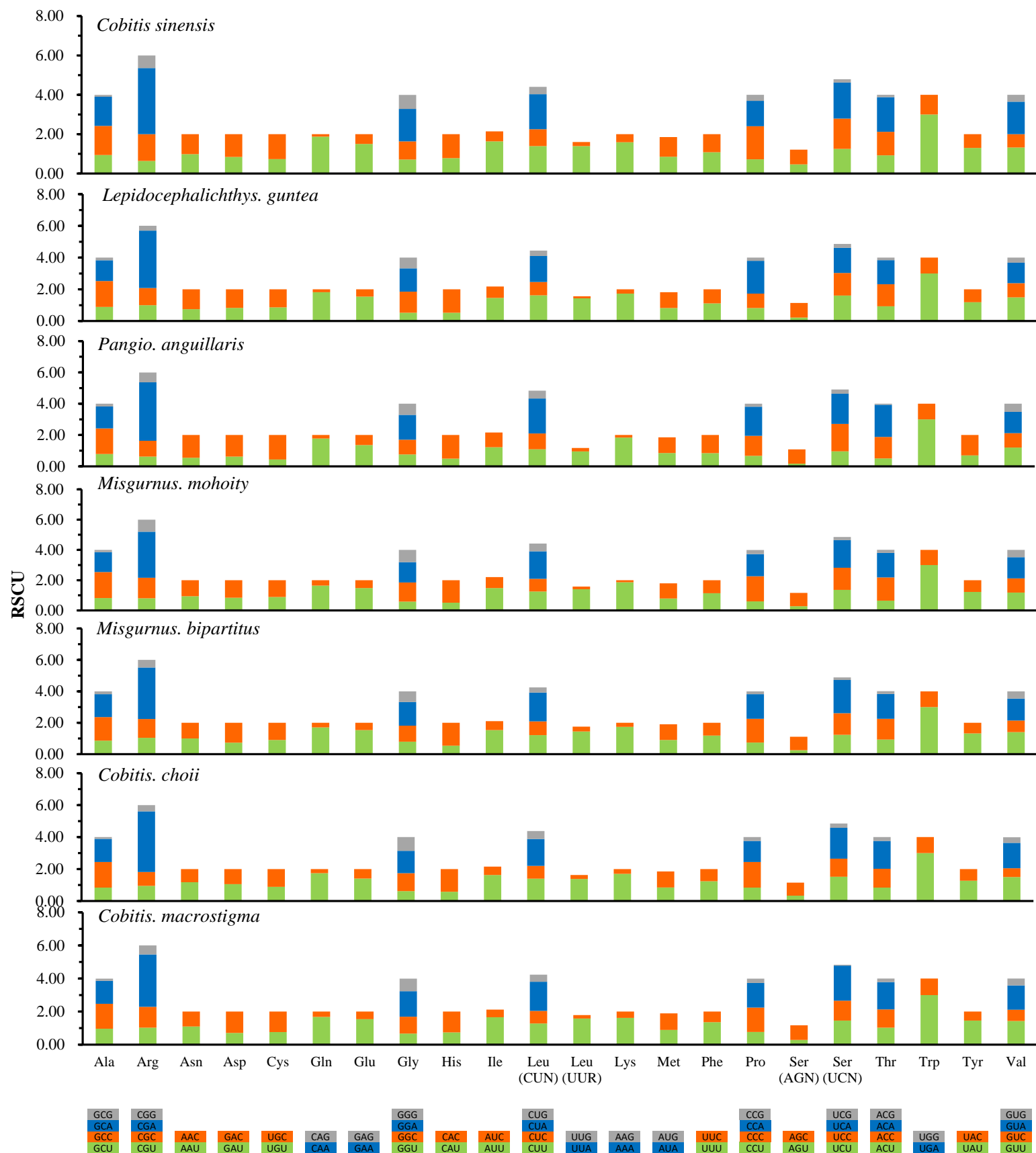

Supplement: Supplementary file 5 — Additional file 5: Figure S1. Codon distribution (A) and relative synonymous codon usage (B) of PCGs in the 58 Cobitinae mitogenomes. CDpT = codons per thousand codons. [file 12864_2020_7360_MOESM5_ESM.pdf]

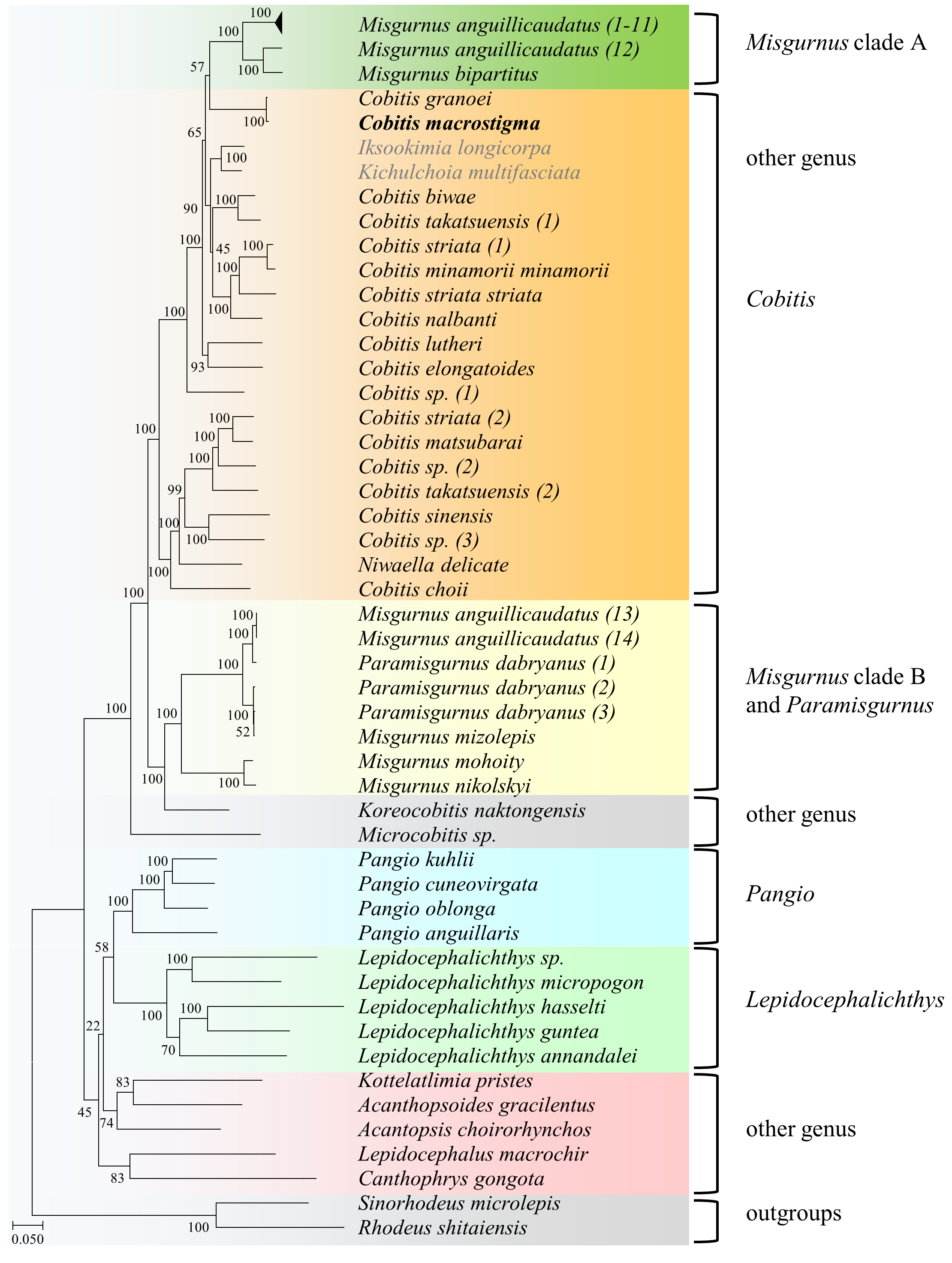

Supplement: Supplementary file 6 — Additional file 6: Figure S2. Phylogenetic tree constructed by ML methods, based on 13 PCGs of 58 Cobitinae mitogenomes. Sinorhodeus microlepis and Rhodeus shitaiensis were chosen as outgroups. Node numbers represent the bootstrap value. [file 12864_2020_7360_MOESM6_ESM.tif]
